# Correction: Opponent learning with different representations in the cortico-basal ganglia pathways can develop obsession-compulsion cycle

**DOI:** 10.1371/journal.pcbi.1011338

**Published:** 2023-07-24

**Authors:** The PLOS Computational Biology Staff

## Notice of republication

An incorrect version of Revision 1, Author Response file was published in error. The publisher apologizes for this error. This article was republished on 10^th^ July 2023 to correct for this error. Please download this article again to view the correct version.
