# Supplementary material for: Opponent learning with different representations in the cortico-basal ganglia pathways can develop obsession-compulsion cycle
Source: PLoS Comput Biol. 2023 Jun 15;19(6):e1011206. doi: 10.1371/journal.pcbi.1011206 (PMC10306209; doi:10.1371/journal.pcbi.1011206)
Supplement: Attachment — Submitted filename: ocd2_responses_to_review_comments_v50.pdf [file pcbi.1011206.s002.pdf]

Response to the review comments

We thank the reviewers very much for giving us the insightful comments. Here we attach our responses. The review comments are in black, and our responses are in blue.

Contents

Response to Reviewer #1's comments : page 2-
Response to Reviewer #2's comments : page 17-
Response to Reviewer #3's comments : page 31-

Notes

- There are similar/related comments by multiple reviewers, but **we respond to each comment of each reviewer without referring to our response to other reviewer's comment**. So there are considerable redundancy between our responses to different reviewers.

- In our responses to the major comments, we attach the primarily corresponding parts of the revised manuscript including figures. The **line numbers mentioned in this document correspond to those in the "Revised manuscript with Changes Highlighted"** (with figures embedded) rather than those in the clean "Manuscript" file (without figures).

List of the main modifications

(Please see our response to the corresponding comment in the following pages for details; as for i) and ii) below, summaries with figures are included in the cover letter)

i) In response to Reviewer 1's point 14 (R1.14), R2.4, and R3.1, we conducted new simulations of the delayed feedback task in Sakai et al., (*Cell Rep* 2022).

ii) In response to R2.4 and R3.4, we analyzed the public data of Gillan et al. (*eLife* 2016).

iii) As for R3.2 (scaling of SR vector), as we set discount factor 0.5, the SR vector's norm^2 was upper-bounded at $(1+0.5+0.5^2...)^2=4$, and since we varied learning rates with $\leq$0.1, effective learning rate was $\leq$0.4, and did not exceed 1. Value diverge was due to the opponent setting, as it occurs also in IR+IR.

iv) In response to R1.12 and R3.3, we fitted the choice of the neutral SR+IR agent in the two-stage task, finding that its model-based weight (w) only tended to differ from the case of the opponent SR+IR. Healthy subjects may thus be modeled by the SR-only, but not the neutral SR+IR, agent, as we did.

v) In response to R1.1, R2.1, and other comments, we have added psychological explanations on how the models relate to OCD behaviors, including the suggested existence of pure compulsion.

Reviewer #1:

Here, the authors attempt to reconcile two current theories underlying obsessive-compulsion - the impairment of the model-based system with a shorter memory trace for negative (than positive) PE. They simulated a model with an agent containing an SR (model-based) and IR (model-free) system, and adjusted its learning based on negative and positive PE at different rates. They found that if the agent's SR system learned primarily from positive PEs, while the IR system learned primarily from negative PEs, the agent would result in an increased looping of the obsession-compulsion states. Further, they simulated the behavior of this (SR+IR) agent completing the two-step task, vs an agent that only has the SR system. They found that the SR+IR agent had lower model-based control than the SR agent. Overall, the authors suggest that the opponent learning of positive PEs for SR and negative PEs for IR may underlie obsession-compulsion.

I thank the authors for an interesting manuscript. I thought the work was quite intriguing, and is indeed a step towards delineating mechanisms underlying OCD, extending both the goal-directed theory and the memory trace theory. I also appreciate the many details that went into the work. I do think the manuscript could work with some tweaks to the grammar and overall rewriting to increase the readability. The results could work with better signposting on the motivation of the analysis - especially since the methods are at the end, it requires more context. Finally, there are a lot of dense concepts which are dropped with minimal explanation and referred to back and forth - where further elaboration/explanation would help to hone in on the key messages put forward. More specific comments are as follows:

Major points

R1.1

1) The introduction starts off very curt, stating the two theories under investigation of the paper, but does not really explain why the theories are related to OCD. How is impaired goal-directed control related to OCD symptoms? Perhaps some reference to dual system theories might help to set the scene. How does having a short memory trace for negative PEs also contribute to OCD? How does it affect learning and therefore behavior (e.g., lower punishment learning, therefore make more wrong actions)?

We have added explanations on OCD and the dual process theories at the beginning of the Introduction, and psychological interpretation on how appetitive SR / longer memory trace for positive RPEs could relate to OCD symptoms at the last part of the Introduction.

<Descriptions in the revised manuscript>

Introduction

(Lines 91-99)
Obsessive-compulsive disorder (OCD) is one of the major psychiatric disorders diagnosed in 2.5%-3% of the population [1], and is characterized as an enhanced cycle of obsessive thought, e.g., whether the door was locked, and compulsive action, e.g., checking door lock. It remains elusive why such an apparently maladaptive behavior could be enhanced. Dual process theories and their advanced forms [2-4] suggest that humans use two control systems, one flexible yet costly goal-directed/model-based system, where the "model" refers to the internal model of the environments (state transitions and rewards/punishments), and another inflexible yet costless habitual/model-free system. A prevailing suggestion based on these theories is that impairment of the goal-directed/model-based system relates to OCD [1, 5-7], potentially explaining its apparently irrational behavior.
(Lines 164-168)
Psychologically, in the appetitive SR + aversive IR agent, reinforcing effects of the pleasantness of a relief from obsession (though not necessarily consciously perceived) operate more generally or widely (or in longer time scales due to the similarity between SR and eligibility trace) than reinforcing effects of the unpleasantness of a stay at obsession. In other words, this agent is characterized by (over)generalization of the pleasantness of a relief from obsession.

R1.2

2) It would be more cohesive if SR and IR were introduced in parallel to model-based/model-free - currently there is a link between SR and model-based control, but IR link to model-free only comes into the last paragraph

We have added a link to model-free RL at the place where we introduce IR.

<Description in the revised manuscript>

Introduction

(Lines 123-124)
SR and more classical individual (punctate) representation (IR), the latter of which can implement model-free control

R1.3

3) "Moreover, we also expected that fitting of behavior of the agent with combined appetitive SR- and aversive IR-based systems could potentially result in smaller weights of model-based control than the agent with SR-based system only in the two-stage decision task" - isn't this automatically expected because SR, as the point was made above, is a model-based system, and thus would have greater model-based control vs a SR-IR (includes model-free) system?

We admit that it was not quite surprising that the estimated model-based weight parameter (w) for the opponent SR+IR agent was smaller than the estimated w for the SR-only agent, and in fact we had expected it as we described in the Introduction. However, even though a reduction of estimated w was expected, it was not possible to expect how much reduction occurred (in particular, whether it could

be comparable to the observed reduction in OCD patients). We think our result that the opponent SR+IR agent was fitted with rather small values of *w* (Figure R1.3), despite being half made of SR-based learner, is still meaningful in this regard. We have added a note on this to the Results. Please see also our response to your point 12, which is related to this issue.

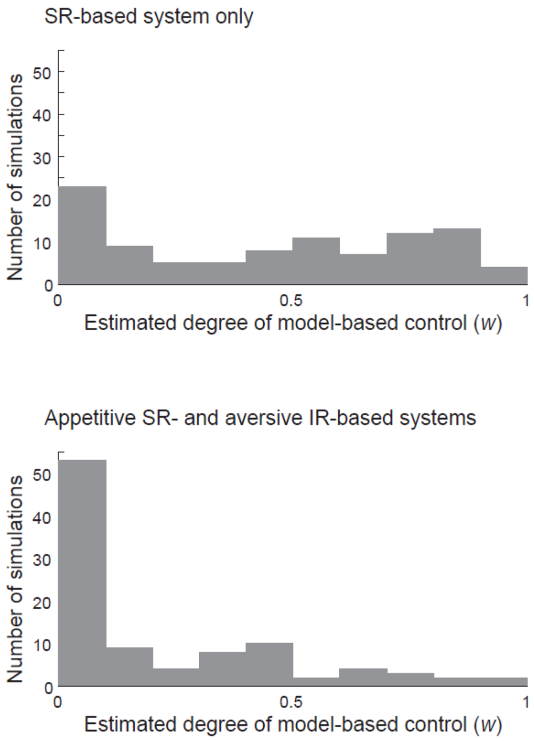

Figure R1.3 (Figure 6A,B in the manuscript)

<Description in the revised manuscript>

> ***Results***
>
> (Lines 552-554)
>
> This reduction of w was rather prominent, even though the appetitive SR + aversive IR agent was half made of SR learner, and we think it could thus potentially explain the reduction of *w* observed in OCD patients [7].

R1.4

4) It would help if Figure 1A included the outcomes, to contrast that the IR has a direct representation of A1 to final outcome.

Thank you for the suggestion. We have added how outcome expectation (action value) is given in SR vs IR to Figure 1A.

<Description/figure in the revised manuscript>

> ***Figure 1A***
>
> (shown in the right)
>
> ***Legend***
>
> (Lines 201-208)
>
> ...Value of A_1 is given by the dot product of the vector of occupancy of each action (x_1 x_2 x_3 x_4 x_5) and a weight vector (w_1 w_2 w_3 w_4 w_5), which is updated by reward prediction errors (RPEs). By contrast, in IR, action A_1 is represented just by itself, separately from other actions. Value of A_1, $Q(A_1)$, is directly updated by RPEs.

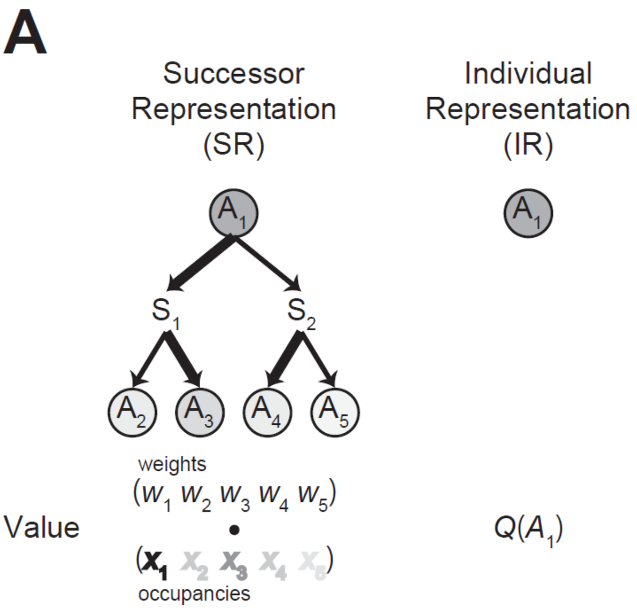

R1.5

5) "whereas eligibility-trace imbalance per se would not bias the estimation of the degree of model-based control." - so is the point that it requires both the model-based and model-free system, in combination with a eligibility-trace imbalance, to underlie OCD symptoms (vs just an imbalanced trace in the model-based system)? This seems to a main finding, and is not very well motivated in the introduction, so the analysis/results come as a surprise.

The separate eligibility trace RL model developed in Sakai et al. (2022 Cell Rep) was purely model-free, and the eligibility trace in the hybrid model-based & model-free model developed in Daw et al., (2011 Neuron) was incorporated in the model-free part of the model. Given these, the result that modification of the eligibility trace of the Daw et al.'s model from balanced to imbalanced one did not affect estimation of the weight of model-based control (w) was not quite surprising, and thus we in fact expected it as mentioned in the Introduction. However, the result was not trivial, given the complexity of the process of RL model fitting. Please see also our response to your point 11 below.

R1.6

6) The description of the environmental model was fairly clear, although in its figure, the loop in the obsession state to itself is confusing because the "other" label is very close to it

The black loop from the obsession state to itself close to the "other" indeed belongs to the "other" action (written in black). Specifically, if the agent takes the "other" action at the obsession state, the agent transitions to the relief state with probability 0.1 and transitions back to (i.e., stays at) the obsession state with probability 0.9. There is another, red loop (from the obsession state to itself) that belongs to the "compulsion" action (written in red).

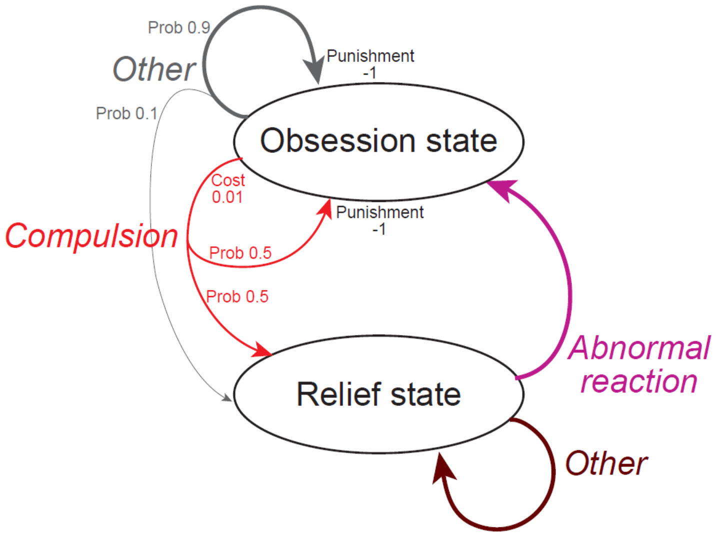

R1.7

7) Behavior of the dual-system agent in the environmental model - authors refer to Figure 3 indicating what the figure shows generally, but not its trends i.e. "Figure 3A-top shows an example of the time evolution of the difference in the (integrated) action values of "abnormal reaction" and "other" at the relief state (Qabnormal − Qother@relief)." but instead could described as "proportion of obsession

state stays low/below threshold, indicating that the agent minimized selection...etc, etc". I think it would help in actually describing the results in the text, because with the current descriptions it does not tell me anything except that I have to refer to the figures. Also the bottom Figure 3 y-axes should indicate it is the average of the middle one- it is a bit misleading to have them the same y axis label.

We have revised (added actual explanations to) the main text according to your suggestion, and also modified the label of the bottom panels of Figure 3A and also others ("Average" was added).

<Description/figure in the revised manuscript>

Results

(Lines 316-322)

Figure 3A-top shows an example of the time evolution of the difference in the (integrated) action values of "abnormal reaction" and "other" at the relief state ($Q_{\text{abnormal}} - Q_{\text{other@relief}}$). The difference became negative, indicating that the abnormal reaction became unlikely to be taken. Figure 3A-middle shows the moving average of the proportion that the agent was at the obsession state in the same example simulation, and Figure 3A-bottom shows the average of such a moving-average proportion across 100 simulations. The proportion became, and remained to be, low.

Figure3A-bottom

(shown in the right)

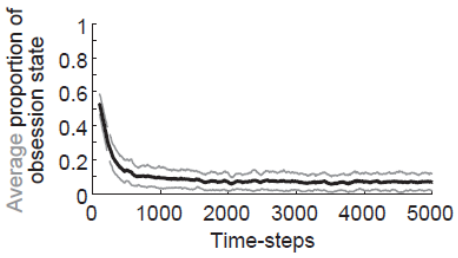

R1.8

8) Similar comments as above to "Figure 3D show the results."

As for this place, explanation of the contents of the figures is actually done in the subsequent main text, and so we have deleted the sentence "Figure 3D show the results."

<Description in the revised manuscript>

Results

(Lines 336-339)

As shown in Figure 3D-top, different from the previous cases, the $Q_{\text{abnormal}} - Q_{\text{other@relief}}$ difference did not smoothly decrease but remained to fluctuate and sometimes even became positive, indicating that this agent continued to take the "abnormal reaction" and transition to the obsession state rather frequently, as actually appeared in Figure 3D-middle,bottom.

R1.9

9) In Figure 6, y-axes are missing, and it would nice to add a distribution curve since the authors are comparing distributions

We have added a label for y-axes: "Number of simulations". As for the addition of curves, because the obtained distributions (in particular (A) and (B)) do not have simple Gaussian or exponential shapes, it is not sure what functions should be used (and also adding curves could potentially bias observation of the original distributions), and so we have not added curves.

<Figure in the revised manuscript>

Figure6A-left

(shown in the right)

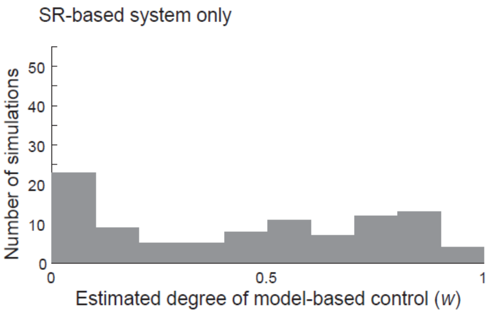

R1.10

10) "As shown in the left panels of Figure 6A,B, the distribution of the estimated w for the agent with appetitive SR- and aversive IR-based systems (Figure 6B) was smaller than that for the agent with SR-based system only (Figure 6A)." Consider rephrasing "smaller distribution"; does this refer to the variance/mean peak? Looks like Figure 6B ranges from 0 to 1, similar to Figure 6A.

We meant to refer to the difference in the overall distributions, compared by the Wilcoxon rank sum test. We have rephrased the description.

<Description in the revised manuscript>

Results

(Lines 550-552)

the estimated *w* for the appetitive SR + aversive IR agent (Figure 6B) was generally smaller (as a distribution) than that for the SR-only agent (Figure 6A) (Wilcoxon rank sum test, $p = 1.04 \times 10^{-7}$)

R1.11

11) "In contrast, fitting of choices generated by the original RL model with balanced eligibility trace ($\lambda = 0.4$ for both positive and negative RPEs) and those generated by a modified model with imbalance trace ($\lambda = 0.4$ and 0 for positive and negative RPEs, respectively), both of which had the same degree of model-based control (w = 0.5), did not result in smaller estimated w in the latter, as shown in the left panels of Figure 6C,D" - This analysis needs to be more clearly motivated. What is the point of testing the eligibility trace difference after the SR-IR system one, and what is expected from this? Also a bit unclear on if both models were specified with the same degree of w, why would it possibly result in differing w?

The reason why we estimated the weight of model-based control *w* for the models with balanced and imbalanced eligibility traces was as follows. If *w* did not differ between these two models, and if the OCD patients examined in Sakai et al. indeed had imbalanced trace as suggested in that paper, the

patients should not largely overlap with OCD patients suggested to show impaired model-based control in other studies, but it seems not very likely (given that Sakai et al. did not recruit some specific subtypes of OCD patients), and therefore we can infer that the OCD patients examined in Sakai et al. may not actually have imbalanced trace but instead have opponent SR+IR (or model-based + model-free) controls. We have added a description about this in the Results.

<Description in the revised manuscript>

> ***Results***
> (Lines 559-563)
> this result indicates a possibility that the OCD patients who were suggested to have imbalanced eligibility trace [10] might not actually have imbalanced trace but instead have opponent SR+IR structure and overlap with previously examined OCD populations who showed impairment of model-based control [7].

As for your last point, even though the modified model with imbalanced trace had the same value of w (0.5) as the original model with balanced trace, because it was fitting of a model by a different model, estimation of w (as well as other parameters) can be spuriously biased, although it was not quite likely and thus we in fact expected that imbalance trace would not affect estimation of w (as mentioned in the Introduction).

R1.12

12) I wonder if it would be a fairer comparison to contrast the appetitive SR and aversive IR model with other versions of the SR-IR model (e.g., differing with pos/neg PE learning) like the models in the paragraph above. This could highlight the contribution of the appetitive/aversive component of the SR-IR model, which presumably is the main aspect that underlies the OC cycle.

We had not conducted estimation of w for the neutral SR+IR agent (α_{SR+}, α_{SR-}, α_{IR+}, α_{IR-}) = (0.15, 0.15, 0.15, 0.15) upon original submission, but we have now conducted it and found that the neutral SR+IR agent was also fitted with small values of w that did not drastically differ from those for the opponent SR+IR agent (Figure R1.12). Thus, from the estimation of w only, we cannot say which of the opponent SR+IR or the neutral SR+IR can be a better model for OCD. One support for the opponent SR+IR over the neutral SR+IR is our simulation result that only the former developed enhanced obsession-compulsion cycle (and because of this, we originally only estimated w for the opponent SR+IR). Empirically, the opponent SR+IR can be distinguished from the neutral SR+IR in the two-stage task by examining if there exists an asymmetry in the common-vs-rare difference in the proportion of "stay" between after reward and after no-reward.

Notably, the abovementioned result that the neutral SR+IR agent was fitted by rather low values of *w* (while the SR-only agent was fitted by relatively large values of *w*) gives a rationale for modeling healthy subjects by the SR-only agent rather than by the neutral SR+IR agent. We consider, however, that healthy people would not always use SR(model)-based learning but may also use IR-based learning, or even appetitive SR- and aversive IR-based learning as it was shown to be advantageous in certain dynamic environments (Morita et al., 2023, *eNeuro*). Healthy people might be able to strongly rely on SR(model)-based system when appropriate while OCD patients might have an impairment in such an arbitration of behavioral controls, potentially in line with a previous suggestion of a weakness of the arbitration mechanism in OCD (Gruner et al., 2016, *Neuroscientist*).

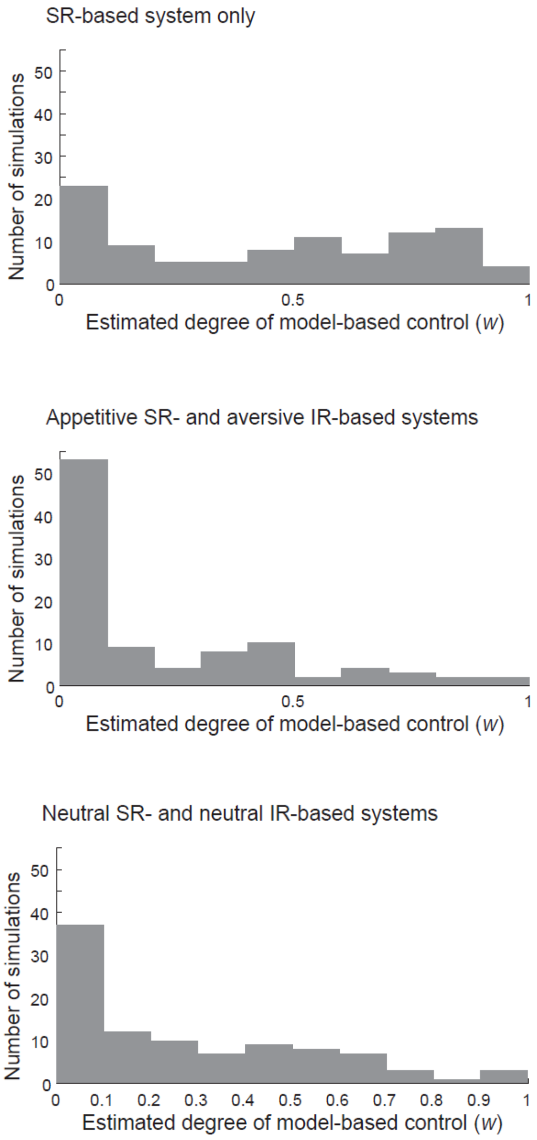

Figure R1.12 (Figure 6A,B,E in the manuscript)

<Descriptions/figures in the revised manuscript>

Results

(Lines 564-570)

We further conducted the fitting analysis for the neutral SR + IR agent. As shown in the left panel of Figure 6E, the estimated *w* was typically low, and its distribution did not drastically differ from the case of the appetitive SR + aversive IR agent, although there was a tendency (Wilcoxon rank sum test, $p = 0.05857$). Thus, from the estimation of the weight of model-based control (*w*) only, we cannot say that the appetitive SR + aversive IR agent is a better model of OCD patients than the neutral SR + IR agent, while such a claim was supported by the abovementioned simulation results (Figure 3 and 4H).

(Lines 600-614)

Therefore, whether the appetitive SR + aversive IR agent or the neutral SR+IR agent better describes OCD could be distinguished by examining whether there exists such an asymmetry between after reward and after no-reward in the common-vs-rare difference in the "stay" proportion.

Discussion

(Lines 809-817)

We modeled the behavior of healthy subjects in the two-stage task by the SR-only agent rather than by the neutral SR+IR agent. This could be justified by the results that the choices of the neutral SR+IR agent were fitted with rather low weights of model-based control (*w*) while those of the SR-only agent were fitted by relatively large values of *w* (Figure 6A,E, left panels). We consider, however, that healthy people would not always use SR(model)-based learning but may also use IR-based learning, or even appetitive SR- and aversive IR-based learning when it is advantageous. Healthy people might be able to strongly rely on SR(model)-based system when appropriate while OCD patients might have an

impairment in such an arbitration of behavioral control, potentially in line with a previous suggestion [44].

Figure 6E

(shown in the bottom panel of Figure R1.12 in the previous page)

Legend

(Lines 635-637)

Results for the neutral SR + IR agent ((α_{SR+}, α_{SR-}, α_{IR+}, α_{IR-}) = (0.15, 0.15, 0.15, 0.15)). Configurations are the same those in (A-D), with the left panel showing the distribution of w across 97 out of 100 simulations.

R1.13

13) "We further analyzed the choice pattern of each type of agent. The agent with appetitive SR and aversive IR-based systems made a model-based-type choice, taking into account whether the occurred state transition was the common type or rare type, after reward was obtained but much less so after reward was not obtained (Figure 6B, right panel). In contrast, the SR-only agent, as well as the agents with balanced or imbalanced eligibility trace, made a model-based-type choice regardless of whether reward was obtained at the previous trial (Figure 6A,C,D, right panels)." This paragraph requires rephrasing and actual explanation of what the pattern of choices mean. Model-based behavior dominates in Figures 6A, C and D vs the influence of model-free in Figure 6B- it is inaccurate to call it "making model based choice", because it is not about a single choice but stay probability over all trials.

We have rephrased these sentences, and added more detailed explanation.

<Description in the revised manuscript>

Results

(Lines 571-600)

We analyzed the choice pattern of each type of agent, focusing on how much the agent's choices (apparently) took into account whether the occurred state transition was the common type or the rare type. Specifically, we focused on the degree with which the agent made more "stay" (i.e., chose the same first-stage option) after reward was obtained in the case of common transition as compared to rare transition, and also made less stays after no reward was obtained in the case of common, as compared to rare, transition. The appetitive SR + aversive IR agent showed a smaller common-vs-rare difference in the "stay" proportion after no-reward than after reward (Figure 6B, right panel), as naturally expected from the combination of appetitive/aversive learning and SR/IR. In contrast, in the SR-only agent, the neutral SR+IR agent, as well as the agents with balanced or imbalanced eligibility trace, the common-vs-rare difference in the "stay" proportion was similar between after reward and after no-reward (Figure 6A,C,D,E right panels).

R1.14

14) Overall, the study presents some convincing evidence to the appetitive SR and aversive IR role underlying the OC cycle, particularly the demonstration of how the loop gets stuck in the obsessional-compulsion states. However I think there could be more comparisons with the models applied to the two step task, the difference between and SR system only and the appetitive/aversive IR system seem to have many components that would contribute to the difference in behavior, and it would be clearer to delineate them.

In response to this your comment and also Reviewer 3's point 1 (R3.1), we have newly conducted simulations of the delayed feedback task of Sakai et al., 2022, and confirmed that our dual-system agents could explain the experimental results of Sakai et al., 2022. Specifically, the appetitive SR + aversive IR agent showed a specific impairment in learning of delayed feedbacks (Figure R1.14B-top), and fitting of its choices by the separate eligibility trace model of Sakai et al. resulted in a shorter trace for negative PREs (Figure R1.14B-bottom), as compared to the SR-only agent.

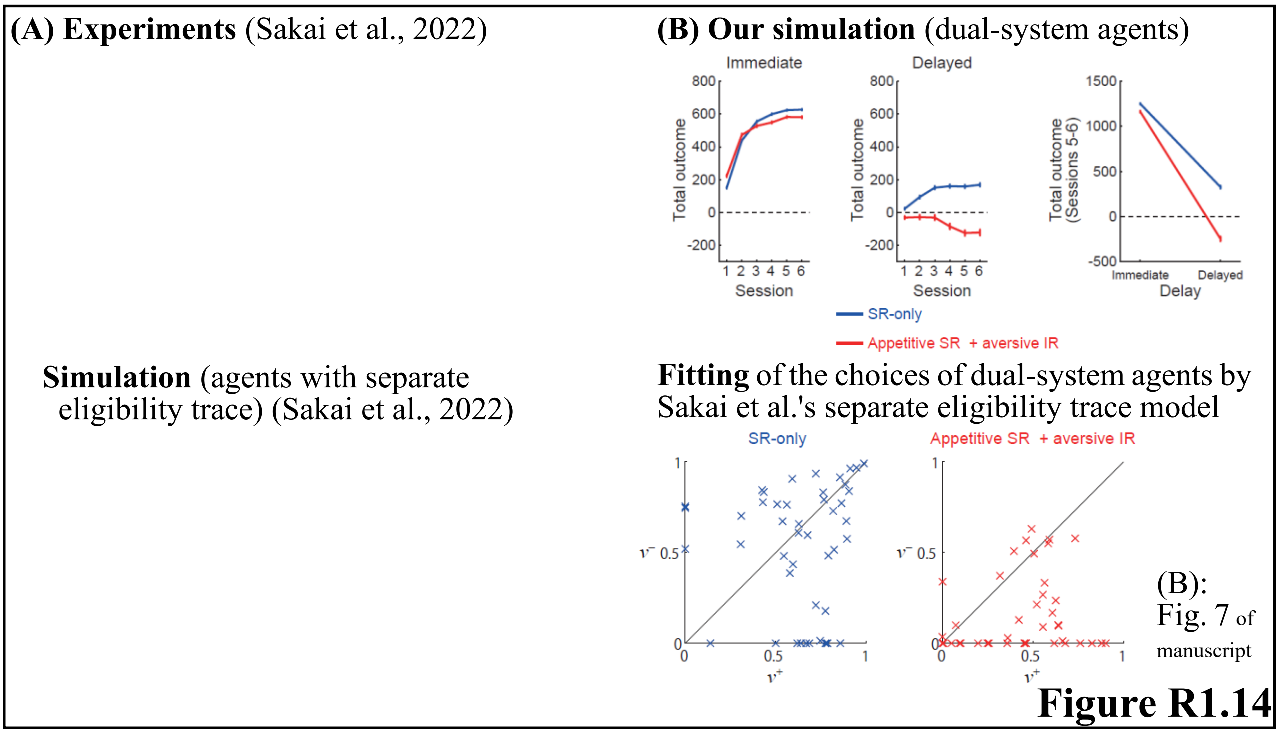

Figure R1.14

<Descriptions/figures in the revised manuscript>

Results

(Lines 639-661)

Behavior of the dual-system agents in the delayed feedback task

We examined whether the observed differential behaviors of OCD patients and HCs in a delayed feedback task [10] could be explained by our dual-system agents. In the task, a pair of stimuli were presented, and subjects were required to choose one of them. Some stimuli caused immediate positive or negative feedback (monetary gain or loss) in the same trial, while other stimuli caused delayed feedback three trials later. Compared to HCs, OCD patients showed intact learning of stimuli causing immediate feedback but impaired learning of stimuli with delayed feedback (Figure 3B,C of [10]), and

this pattern was reproduced by models with balanced and imbalanced eligibility traces (Figure 3E,F of [10]). We simulated execution of this task by the dual-system agents, in which actions of choosing each stimulus were represented by estimated discounted cumulative occupancies of successor states defined based on presented feedback (see the Materials and Methods for details). As shown in Figure 7A,B, the appetitive SR + aversive IR agent (α_{SR+}, α_{SR-}, α_{IR+}, α_{IR-}) = (0.018, 0.002, 0.002, 0.018), as compared to the SR-only agent (α_{SR+}, α_{SR-}, α_{IR+}, α_{IR-}) = (0.02, 0.02, 0, 0), showed a particular impairment in learning of delayed feedback, largely reproducing the pattern observed in the experiment [10].

We further fitted the choices of these two types of dual-system agents by the separate eligibility trace actor-critic model considered in [10]. As a result, the choices of the SR-only agent were in large part fitted by the model with large eligibility traces for both positive and negative RPEs whereas the choices of the appetitive SR + aversive IR agent were in large part fitted by the model with a larger trace for positive than negative RPEs (Figure 7C), as we expected from the similarity between the eligibility trace and SR. These results indicate that the observed behaviors of OCD patients and HCs in the delayed feedback task [10] could be explained by the appetitive SR + aversive IR agent and the SR-only agent, respectively.

Figure 7

(shown in the right)

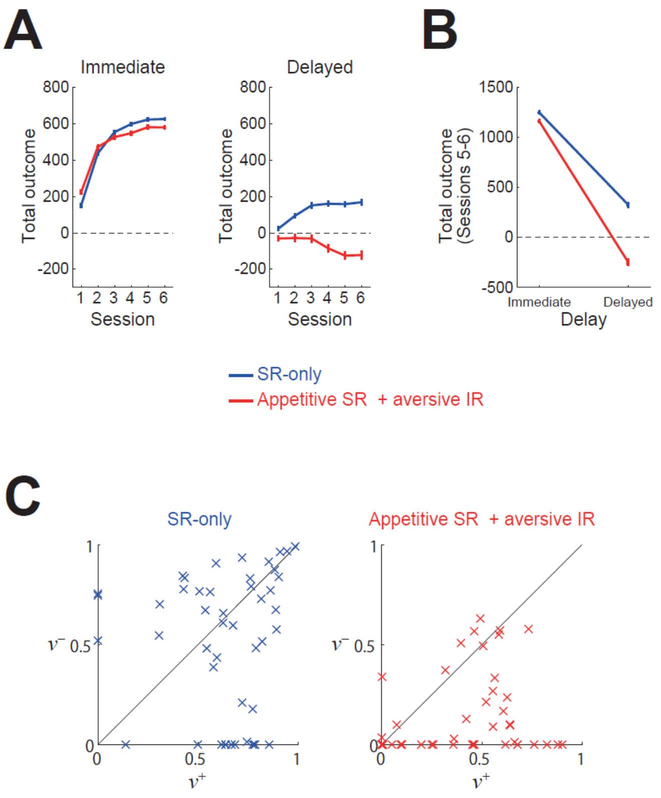

Legend

(Lines 701-711)

Behavior of the dual-system agents in the delayed feedback task examined in [10]. **(A)** Total obtained outcome (feedback) from stimuli causing immediate (left) or delayed feedback (right) in each session, averaged across 1000 simulations, for the appetitive SR + aversive IR agent (red) and the SR-only agent (blue). The error-bars indicate ±SEM. **(B)** Total obtained outcome (feedback) in sessions 5 and 6, averaged across 1000 simulations, from stimuli with immediate or delayed feedback for the two types of agents. The error-bars indicate ±SEM. **(C)** Results of fitting of the choices of the two types of agents (47 and 45 out of 50 simulations for each, separately conducted from those shown in (B)) by the separate eligibility trace actor-critic model considered in [10]. The horizontal and vertical axes indicate the estimated parameters v^+ and v^- (decay time scale of the trace for positive and negative RPEs), respectively.

Materials & Methods

(Lines 1225-1279)

Simulation and fitting of the delayed feedback task

We simulated execution of the delayed feedback task [10] by the dual-system agents. There were eight stimuli, which caused +40, +10, −10, or −40 immediate feedback (denoted as $+40^{im}$ etc) or +40, +10, −10, or −40 delayed feedback three trials later (denoted as $+40^{de}$ etc). The task consisted of six sessions, and in each session, each of six pairs of stimuli (($+10^{im}$, $+40^{im}$), ($+10^{de}$, $+40^{de}$), (-10^{im}, -40^{im}), (-10de, -40de), ($+10^{im}$, $+40^{de}$), (-10^{im}, -40de)) was presented ten times and each of ten pairs (($+40^{im}$, $+40^{de}$), ($+10^{im}$, $+10^{de}$), (-10^{im}, -10de), (-40^{im}, -40de), ($+10^{de}$, $+40^{im}$), (-10de, -40^{im}), ($+10^{im}$, -10^{im}),

($+40^{\mathrm{im}}$, -40^{im}), ($+10^{\mathrm{de}}$, -10de), ($+40^{\mathrm{de}}$, -40de)) was presented five times in a pseudorandom order (in the same manner as in the original study [10]).

The IR-based system of the dual-system agent learned the system-specific values of eight actions to choose each of the eight stimuli through updating the value of taken action by RPE multiplied by the learning rate ($\alpha_{\mathrm{IR}+}$ or $\alpha_{\mathrm{IR}-}$ for positive or negative RPE, respectively). The SR-based system represented each action by estimated discounted cumulative occupancies of successor "states". Seven states were defined based on the presented (total) feedback at each trial: S_1: +80, S_2: +50 or +40 or +30, S_3: +20 or +10, S_4: 0, S_5: −10 or −20, S_6: −30 or −40 or −50, and S_7: −80. At each trial k, "trace" of each action A_i (i = 1, .., 8), $T(A_i)$, was updated as

$T(A_i) \leftarrow \gamma T(A_i)$ (if A_i was not taken at trial k) or

$T(A_i) \leftarrow \gamma T(A_i) + 1$ (if A_i was taken at trial k),

where γ was the time discount factor set to 0.75. Then, a column of 8×7 SR matrix **M** that corresponded to the state at trial k (determined by the feedback presented at trial k), S_j, was updated to be equal to the traces for each action:

$\mathbf{M}_{ij} \leftarrow T(A_i)$ (i = 1, .., 8),

after **M** was used for value calculation and value (weight) update (mentioned below). The other columns of **M** remained unchanged. The SR-based system-specific value of A_i (i = 1, .., 8) was given by the dot product of the SR feature vector for A_i, i.e., i-th row of **M**, $\mathbf{M}_{(i)}$, and a weight vector **w**, i.e., $\mathbf{w}\cdot\mathbf{M}_{(i)}$. The weight **w** was updated based on the action A_i that was taken at trial k and RPE δ:

$\mathbf{w} \leftarrow \mathbf{w} + \alpha_{\mathrm{SR}+}\delta\mathbf{M}_{(i)}$ (when $\delta \geq 0$) or

$\mathbf{w} \leftarrow \mathbf{w} + \alpha_{\mathrm{SR}-}\delta\mathbf{M}_{(i)}$ (when $\delta < 0$).

Integrated action values were calculated as the average of the IR- and SR-based system-specific action values. Choice between two actions corresponding to two stimuli presented at each trial was made in the soft-max manner with the degree of exploitation over exploration (inverse temperature) (β) set to 0.25. RPE was calculated as the difference between the (total) feedback and the integrated value of taken action. The IR-based system-specific values for all the actions and all the elements of the weight vector for the SR-based system-specific values (**w**) decayed at a constant rate (0.001), i.e., were multiplied by 0.999 at each trial. The traces for actions $T(A_i)$, the elements of the SR matrix **M**, and the elements of the weight vector **w** were all initialized to 0. The learning rates were set as ($\alpha_{\mathrm{SR}+}$, $\alpha_{\mathrm{SR}-}$, $\alpha_{\mathrm{IR}+}$, $\alpha_{\mathrm{IR}-}$) = (0.02, 0.02, 0, 0) or (0.018, 0.002, 0.002, 0.018) for the SR-only agent or the appetitive SR + aversive IR agent, respectively. We conducted 1000 simulations for each of the two types of agents, and calculated the averages and the SEMs.

We also conducted fitting of the choices of these two types of dual-system agents (50 simulations for each, separately conducted from the abovementioned 1000 simulations) by the separate eligibility trace actor-critic model considered in [10]. This model had four free parameters: a (learning rate), b (inverse temperature for choice), and v^+ and v^- (decay time scale of the trace for positive and negative RPEs, respectively). Since a, v^+, and v^- should normally be between 0 and 1, these variables were transformed into atanh($2x$−1), where x represents a, v^+, or v^-, in the fitting process so that the range of exploration was effectively bound to [0 1]. For each choice sequence, we explored a set of parameters that maximized the log likelihood [60] by using the "fminsearch" function of MATLAB, with the initial parameter values set as (a, b) = (0.0011, 2.96), which were the medians of estimated parameters of HCs in [10], and v^+, v^- = 0.1, 0.5, or 0.9 (i.e., 3 × 3 = 9 cases were examined) and the 'MaxFunEvals' and 'MaxIter' options of this function set to both 10000. Among the nine cases with different initial values of (v^+, v^-), we selected the cases in which the "fminsearch" function terminated with "exitflag" 1 (meaning normal termination), and if there were any such case(s) (this was true for 47 and 45 simulations for the SR-only and the appetitive SR + aversive IR agent, respectively), we selected a case that gave the maximum log likelihood among them, and plotted the corresponding values of v^+ and v^- in Figure 7C.

R1.15

15) There is some nuance in the expression of OCD that is not fully taken into account of the current model. OCD patients present varying levels of obsessions vs compulsions, e.g. pure obsessional/compulsive OCD. Whereas the current model assumes that it is a cycle of the obsessive state to the compulsion state (to resolve distress), where both states are necessary. I wonder how these other presentations could be accounted for. Another point to raise is that there is a hypothesis specifically borne from the goal-directed work of compulsions (Gillan & Sahakian, Neuropsychopharmacology, 2015), it is suggested that obsessions in OCD might arise as a result of compulsive behavior instead - what do the authors think of this in terms of their current work?

Thank you for pointing out this issue. As you said, the environmental model that we used (developed by Sakai et al., 2022) assumes the obsession-compulsion cycle as an inseparable core of OCD. However, we think that the model could still be compatible with the existence of (apparently) pure compulsion if the "abnormal reaction/obsession" can be subconscious, and also with pure obsession if the "compulsion" is not a motor action but a thought, presumably depending on which part of cortico-basal ganglia circuits has the opponent SR+IR structure. However, the model, at least in its current form, appears not compatible with the suggestion you mentioned that obsession can emerge from compulsion through erroneous reverse inference, and we think that future studies are desired to test these different theories, or possibly integrate them by elaborating the environmental model, which is currently rather simple. We have added these discussion to the Discussion.

<Description in the revised manuscript>

Discussion

(Lines 923-942)

From a more general viewpoint, a potential limitation of our model is that it may not explain a wide range of phenomena associated with OCD. In particular, it has been suggested that OCD is accompanied with various degrees of obsession and compulsion, and there can be pure compulsion (compulsive hoarding can be it [1]) and pure obsession [56] (but see [1]). By contrast, the environmental model that we used, developed by [10], assumes the obsession-compulsion cycle as an inseparable core of OCD. The model in fact assumes that intrusive thoughts universally occur even in healthy people, and what distinguishes OCD is the "abnormal reaction" to such thoughts. Once entering the obsession state, even the SR-only agent is likely to choose the "compulsion" as it should have a larger value than the "other" because the "other" leads to punishment more frequently than the "compulsion". So in this model, OCD is not characterized by an increase in the value/choice-frequency of compulsion. The model could still be compatible with the existence of (apparently) pure compulsion if the "abnormal reaction/obsession" can be subconscious, and also with pure obsession if the "compulsion" is not a motor action but a thought (as implied in [1]), presumably depending on which part of cortico-basal ganglia circuits has the opponent SR+IR structure. However, it has also been suggested that compulsion (rather than obsession or intrusive thoughts) is a key factor of OCD [1] and obsession can emerge from compulsion through erroneous reverse inference [57]. Future studies are desired to test these different theories, or possibly integrate them by elaborating the environmental model, which is currently rather simple. Given that

environmental complexity was suggested [58] to have crucial effects in addiction, which also entails compulsion, future elaboration of models and exploration of parameters are highly desired.

R1.16

16) "Specifically, we could not find how the model-based system estimated the transition probabilities when the frequencies of past occurrence of both types of transitions exactly matched in the original study, and we assumed that the model-based system estimated them as 50% and 50% in such situations" - Not quite sure what the authors mean here - common vs rare transition is 70/30%, and so the occurrence of the transitions should match that as well? In terms of simulations, the transition matrix would start with 50% for all types of transitions and updates trial by trial based on the choices made in the task.

What we tried to mean was that even though the "common (70%)" type of transition was programed to occur with 70% probability, the *actual* frequency that this type occurred, for example, in how many trials from the 1st trial to the 20th trial does not necessarily match 70% (i.e., 14 out of 20 trials) but should vary (10 trials in one case whereas 16 trials in another etc). We have added explanations to this place.

<Description in the revised manuscript>

Materials & Methods

(Lines 1209-1211)

we could not find how the model-based system estimated the transition probabilities when the actual frequencies of past occurrence of both types of transitions exactly matched (for example, both types of transitions occurred 10 trials in the initial 20 trials) in the original study

Minor points

R1.17

17) The authors refer to 'agent' without an article (an/the) throughout the text, it reads a bit weird without one

Thank you. We have added an article to each 'agent'.

R1.18

18) "Specifically, modeling showed that obsession-compulsion cycle can be enhanced in agent having much shorter memory (eligibility) trace for negative than positive prediction errors (PEs), and fitting of behavioral choices suggested that OCD patients indeed had such an imbalance as compared to

healthy controls (HCs), although even HCs tended to show shorter memory trace for negative than positive PEs." This sentence is way too long and is very confusing. Consider splitting it into more sentences.

We have split the sentence into two, with modifications (Lines 104-109).

R1.19

19) "...this possibility explains why human is prone to OCD and even healthy people tended to show shorter memory trace for negative PEs." - should be 'humans are'. More broadly I think this these two conclusions can be phrased better.

In the revised manuscript, we have changed this last part of the Abstract, now discussing the limitation and its possible resolution because it would need to be more focused (Lines 58-61). We have also changed 'human' in a sentence in the Discussion to 'humans' (Line 742).

R1.20

20) "and fitted them by the RL model weighing model-based and model-free controls developed in the original study" - it should just be 'control' (controls can be misinterpreted as healthy controls)

Thank you. We have corrected this (Lines 53-54).

Reviewer #2:

The manuscript describes an interesting integrative model of OCD, that has the potential to explain both clinical phenomena (persistent obsessions) and well-known two-stage task findings, based on the idea that patients' model-based system (or more specifically - SR) learns disproportionately more from positive RPEs, whereas their model-free system learns more from negative RPEs. I applaud the authors for the attempt to integrate previous computational models of OCD – the field is in desperate need of such endeavors. Furthermore, the model is sophisticated and appears to be biologically plausible.

That being said, I believe that several major conceptual, empirical, and style-related issues limit the potential contribution and impact of the manuscript.

R2.1

First, the model is justified chiefly based on some overlapping neurobiological findings concerning different distinctions between direct and indirect pathways of the basal ganglia. Although this seems reasonable, I believe the manuscript/model lacks sufficient intuitive psychological/clinical justification. What is the psychological interpretation of the model? Is it the idea that OCD patients are more likely to start obsessing because they essentially overweight the probability that they will be able to 'exit' the obsessing cycle via compulsions (such that the SR/MB value of entering it is not as negative as it could have been)? Whether this is the core idea, or a different explanation would be more precise– I believe such an explanation must be repeatedly spelled out in the introduction, and the interpretation of the results. Insufficient interpretability is also evident, for example, regarding the finding that the alternative model with 8 'other' actions does not lead to obsessive phenomenology (also, can this result be attributed specifically to the existence of many 'other' actions starting form the relief state or, alternatively, the 'other' actions starting from the obsession state?).

In general, such interpretability is important for two main reasons. First, although this is a computational paper and a computational journal, I believe its conceptual assumptions/conclusions must be interpretable by OCD experts lacking the extensive technical background the current conceptualization requires. Second, spelling out the conceptual assumptions/conclusions will enhance the ability to evaluate the psychological plausibility of the model.

Thank you for pointing out the lack of psychological interpretations of our model. We have added descriptions about them in the Introduction.

<Description in the revised manuscript>

Introduction

(Lines 164-168)

Psychologically, in the appetitive SR + aversive IR agent, reinforcing effects of the pleasantness of a relief from obsession (though not necessarily consciously perceived) operate more generally or widely (or in longer time scales due to the similarity between SR and eligibility trace) than reinforcing effects of the unpleasantness of a stay at obsession. In other words, this agent is characterized by (over)generalization of the pleasantness of a relief from obsession.

Thank you also for the comment about which of the number of other actions at the relief state or that at the obsession state is crucial. We have newly examined two cases in which the number of other actions at the relief and obsession states was 8 and 1 (Figure R2.1-left) or 1 and 8 (Figure R2.1-right). Enhanced obsession-compulsion cycle was developed only in the latter, indicating that the reduced representation of other actions at the relief state is essential for obsession-compulsion. We have added this result and its psychological interpretation.

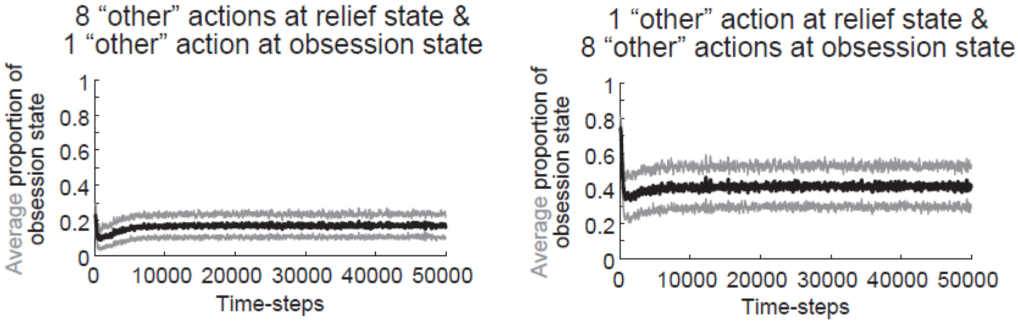

Figure R2.1 (Figure 5F in the manuscript)

<Descriptions/figures in the revised manuscript>

Results

(Lines 501-505)

We further examined the cases where there were eight "other" actions at the relief state and one "other" action at the obsession state (Figure 5F, top) or one "other" action at the relief state and eight "other" actions at the obsession state (Figure 5F, bottom). Enhanced obsession-compulsion cycle was developed only in the latter case.

Figure 5F

(the top and bottom panels are the same as the left and right panels of Figure R2.1 above)

Legend

(Lines 528-532)

Behavior (average ±SD of the moving-average proportion of the obsession state) of the appetitive SR + aversive IR agent with the decay of values/weights in the environment where there were eight "other" actions at the relief state and one "other" action at the obsession state (top) or one "other" action at the relief state and eight "other" actions at the obsession state (bottom).

Discussion

(Lines 746-790)

We have shown that when the number of possible actions other than the abnormal reaction at the relief state was increased, obsession-compulsion cycle was not enhanced. This result suggests that a factor contributing to enhanced obsession-compulsion cycle is that alternative actions are represented in a dimension-reduced manner. Intuitively, this could correspond to a situation where a person concentrates on the emerging intrusive thoughts so much that s/he cannot think of a variety of other things.

R2.2

This raises a second issue that focuses on the specificity of the results to the specific environmental model depicted in Figure 2. A fundamental assumption (or maybe a result) of the model is that the mechanism of obsessive-compulsive behavior is related to a higher probability of having an 'abnormal reaction', or, in other words – a higher probability of experiencing obsessions. This is interesting but also not somewhat controversial. Some classical models of OCD will argue that obsessions (or intrusive thoughts) occur universally and that the core problem in OCD is either a higher probability of staying in the obsessional state or alternatively, a higher probability of using compulsions to exit it. Is there any evidence for such 'behaviors' in the model? For example, does the `OCD agent` use compulsions as a main policy (vs. 'other') to exit the obsessional state? If we examine the two agents (with/without OCD) only in cases/steps in which they have entered the obsessional state – is the OCD agent more likely to use compulsions to exit it? Furthermore, is the 'OCD agent' more prone to 'get stuck' in the obsessions state, or is the general increase in obsession caused by intermittent obsessions-compulsion cycles? More generally - how do excessive learning from positive SR RPEs and reduced learning from positive IR RPEs affect policies starting from the obsession state?

Thank you for pointing out this issue. The environmental model that we used (developed by Sakai et al., 2022) in fact assumes that intrusive thoughts universally occur even in healthy people, but as you said, what distinguishes OCD in the model is the "abnormal reaction" to such thoughts, which automatically leads to the obsession state. Once entering the obsession state, even the SR-only agent is likely to choose the "compulsion" as it has a larger value than the "other" (Figure R2.2) because the "other" leads to punishment more frequently than the "compulsion". So in this model, OCD is not characterized by an increase in the value/choice-frequency of compulsion. The model could still be compatible with the existence of (apparently) pure compulsion if the abnormal reaction and obsession can be subconscious, presumably depending on the part of cortico-basal ganglia circuits that has the opponent SR+IR structure (i.e., if it is the part engaging in subconscious learning and action selection / execution). However, the environmental model that we used is rather simple, and a possible future direction would be to elaborate the model that can describe changes in the policy to take compulsion. We have discussed these in the Discussion.

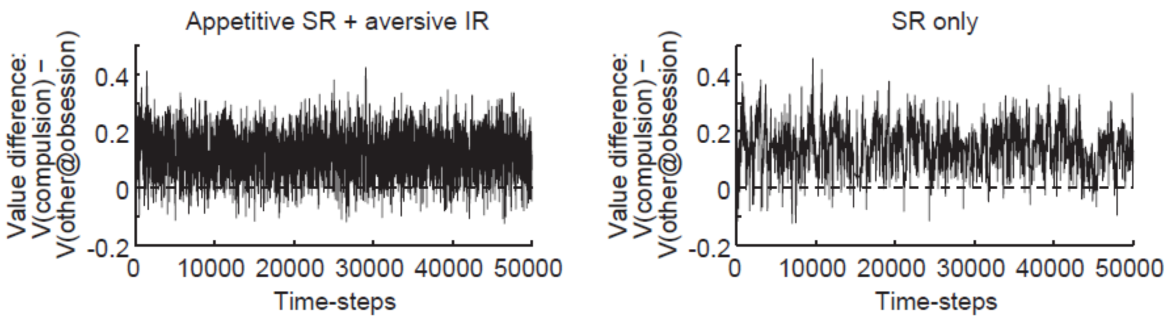

Figure R2.2

<Description in the revised manuscript>

Discussion

(Lines 923-942)

From a more general viewpoint, a potential limitation of our model is that it may not explain a wide range of phenomena associated with OCD. In particular, it has been suggested that OCD is accompanied with various degrees of obsession and compulsion, and there can be pure compulsion (compulsive hoarding can be it [1]) and pure obsession [56] (but see [1]). By contrast, the environmental model that we used, developed by [10], assumes the obsession-compulsion cycle as an inseparable core of OCD. The model in fact assumes that intrusive thoughts universally occur even in healthy people, and what distinguishes OCD is the "abnormal reaction" to such thoughts. Once entering the obsession state, even the SR-only agent is likely to choose the "compulsion" as it should have a larger value than the "other" because the "other" leads to punishment more frequently than the "compulsion". So in this model, OCD is not characterized by an increase in the value/choice-frequency of compulsion. The model could still be compatible with the existence of (apparently) pure compulsion if the "abnormal reaction/obsession" can be subconscious, and also with pure obsession if the "compulsion" is not a motor action but a thought (as implied in [1]), presumably depending on which part of cortico-basal ganglia circuits has the opponent SR+IR structure. However, it has also been suggested that compulsion (rather than obsession or intrusive thoughts) is a key factor of OCD [1] and obsession can emerge from compulsion through erroneous reverse inference [57]. Future studies are desired to test these different theories, or possibly integrate them by elaborating the environmental model, which is currently rather simple. Given that environmental complexity was suggested [58] to have crucial effects in addiction, which also entails compulsion, future elaboration of models and exploration of parameters are highly desired.

R2.3

Third, an interesting result of the simulations is that the probability of experiencing an obsession, even in an `agent with OCD` never surpasses 50%. This raises two questions. First – how plausible is this result? Oftentimes, OCD symptoms can become so dominant in a specific context that patients will tend to experience obsessions and emit a compulsion in close to 100% of the times they find themselves in this context (e.g., while handwashing, or when leaving the house, etc.). Second – what does this entail regarding the theoretical role of the temperature parameter in the model? Isn't it the case that a nearly-deterministic model here will never move to the obsessional state?

As for the first question, we consider that the time scale represented in the model is longer - it would not describe the dynamics within a single episode of handwashing but instead describe intermittent occurrence of such episodes, interleaved by non-compulsive daily behavior.

As for the second question, thank you for the insight. We examined the cases where the inverse temperature β increased from the original value (β = 10). When β increased tenfold (β = 100), development of enhanced obsession-compulsion cycle was on average fairly delayed (Figure R2.3-left), and when it further increased fivefold (β = 500), enhanced cycle was on average not developed within the examined time steps (50000) (Figure R2.3-right), supporting the effect of β that you suggested. We have added these results, with figures.

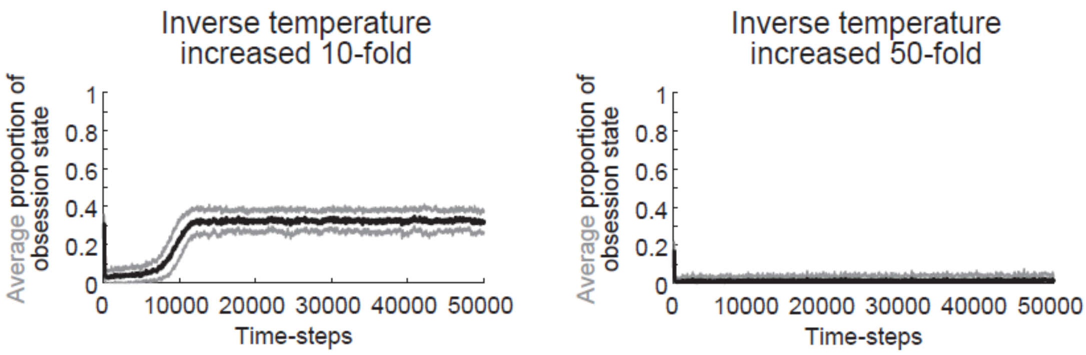

Figure R2.3 (Figure 4I in the manuscript)

<Descriptions/figures in the revised manuscript>

Results

(Lines 440-449)

Notably, because the value of the abnormal reaction was (positive but) lower than the value of the "other" action at the relief state as mentioned above, it seems possible that if the agent's choice policy is nearly deterministic (i.e., the inverse temperature is quite high), the agent would rarely take the abnormal reaction and thereby rarely develop enhanced obsession-compulsion cycle. We examined this possibility. When the inverse temperature increased tenfold (from β = 10 to β = 100), development of enhanced obsession-compulsion cycle was on average fairly delayed (Figure 4I, top), and when it further increased fivefold (β = 500), enhanced cycle was on average not developed within the examined time steps (50000) (Figure 4I, bottom). These results support the abovementioned conjecture about the mechanism and suggest a possible effect of choice exploration/exploitation.

Figure 4I

(the top and bottom panels are the same as the left and right panels of Figure R2.3 above)

Legend

(Lines 474-477)

The average of the moving-average proportion of the obsession state across 100 simulations (black line), presented with ±SD (gray thin lines), of the appetitive SR + aversive IR agent with the decay of values/weights, with the inverse temperature increased tenfold (top) or fifty-fold (bottom: −SD is mostly invisible).

R2.4

Fourth, I believe the ability of the model to provide an alternative explanation for the common finding of reduced model-based learning in the two-step task is intriguing and important. I also applaud the researchers for discussing the limitations of this alternative interpretation vis-à-vis other findings in this literature. I have two questions here. First, can the model also explain findings concerning model-based learning in simple structure learning tasks (e.g., Sharp, Dolan & Eldar, 2021, Psychological Medicine)? Second, it would help the paper dramatically if the authors were able to fit their model to actual two-step task data (some are publicly available, I believe, e.g., https://osf.io/usdgt/)

As for the first question, our dual-agent model has a parameter determining the learning rate for the update of SR matrix according to the TD error of SR features. The impairment of state transition learning in people with compulsivity shown by Sharp et al. could potentially be described in our model by explicitly modulating this parameter, but it is considered to be separate from the opponent learning with different representations that we primarily propose. Nonetheless, we have added a discussion of Sharp et al. at the place where we discussed possible effects of the rigidity of state representation.

<Description in the revised manuscript>

Discussion (Lines 791-792) Empirical work [41] has shown that compulsivity is associated with impairment of state transition learning.

Regarding whether our model can also explain findings in simple structure learning tasks, we have newly simulated the delayed feedback task examined in Sakai et al., (2022, Cell Rep), and confirmed that the appetitive SR + aversive IR agent and the SR-only agent could explain the behaviors of OCD patients and healthy controls, respectively (Figure R2.4.1).

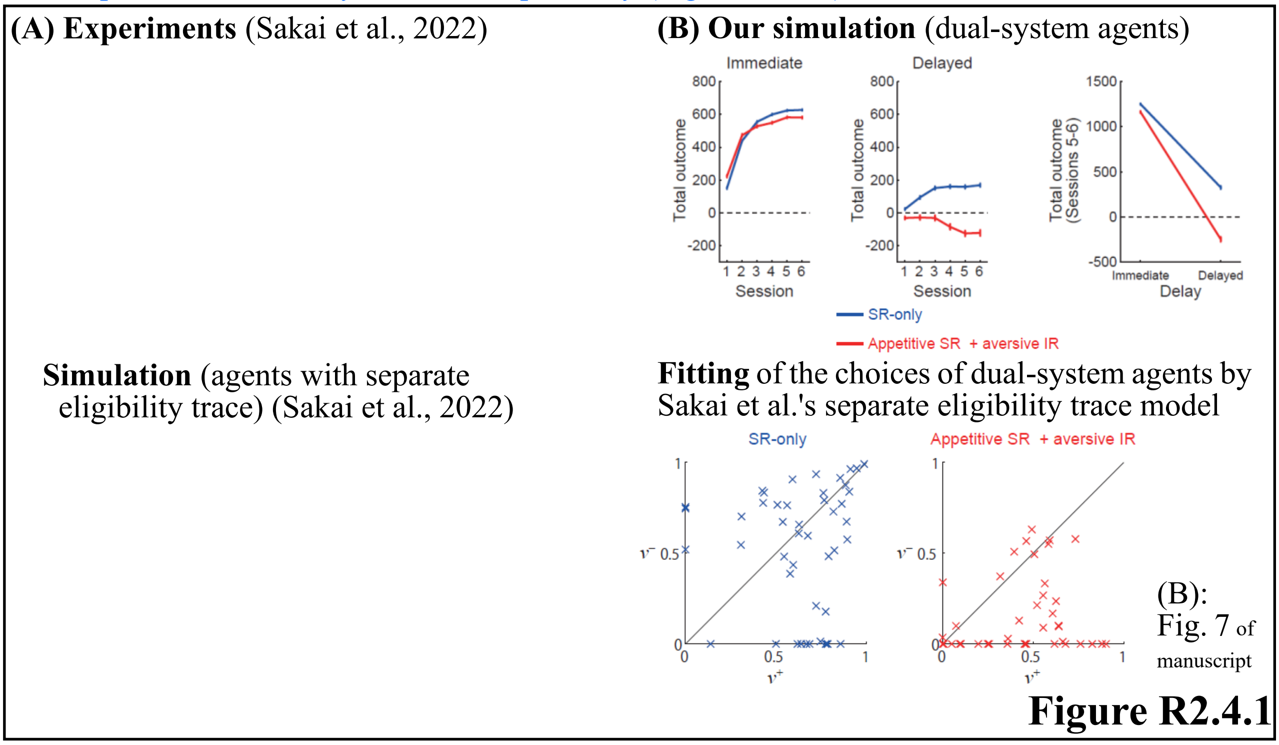

Figure R2.4.1

<Descriptions/figures in the revised manuscript>

Results

(Lines 639-661)

Behavior of the dual-system agents in the delayed feedback task

We examined whether the observed differential behaviors of OCD patients and HCs in a delayed feedback task [10] could be explained by our dual-system agents. In the task, a pair of stimuli were presented, and subjects were required to choose one of them. Some stimuli caused immediate positive or negative feedback (monetary gain or loss) in the same trial, while other stimuli caused delayed feedback three trials later. Compared to HCs, OCD patients showed intact learning of stimuli causing

immediate feedback but impaired learning of stimuli with delayed feedback (Figure 3B,C of [10]), and this pattern was reproduced by models with balanced and imbalanced eligibility traces (Figure 3E,F of [10]). We simulated execution of this task by the dual-system agents, in which actions of choosing each stimulus were represented by estimated discounted cumulative occupancies of successor states defined based on presented feedback (see the Materials and Methods for details). As shown in Figure 7A,B, the appetitive SR + aversive IR agent (α_{SR+}, α_{SR-}, α_{IR+}, α_{IR-}) = (0.018, 0.002, 0.002, 0.018), as compared to the SR-only agent (α_{SR+}, α_{SR-}, α_{IR+}, α_{IR-}) = (0.02, 0.02, 0, 0), showed a particular impairment in learning of delayed feedback, largely reproducing the pattern observed in the experiment [10].

We further fitted the choices of these two types of dual-system agents by the separate eligibility trace actor-critic model considered in [10]. As a result, the choices of the SR-only agent were in large part fitted by the model with large eligibility traces for both positive and negative RPEs whereas the choices of the appetitive SR + aversive IR agent were in large part fitted by the model with a larger trace for positive than negative RPEs (Figure 7C), as we expected from the similarity between the eligibility trace and SR. These results indicate that the observed behaviors of OCD patients and HCs in the delayed feedback task [10] could be explained by the appetitive SR + aversive IR agent and the SR-only agent, respectively.

Figure 7

(shown in the right)

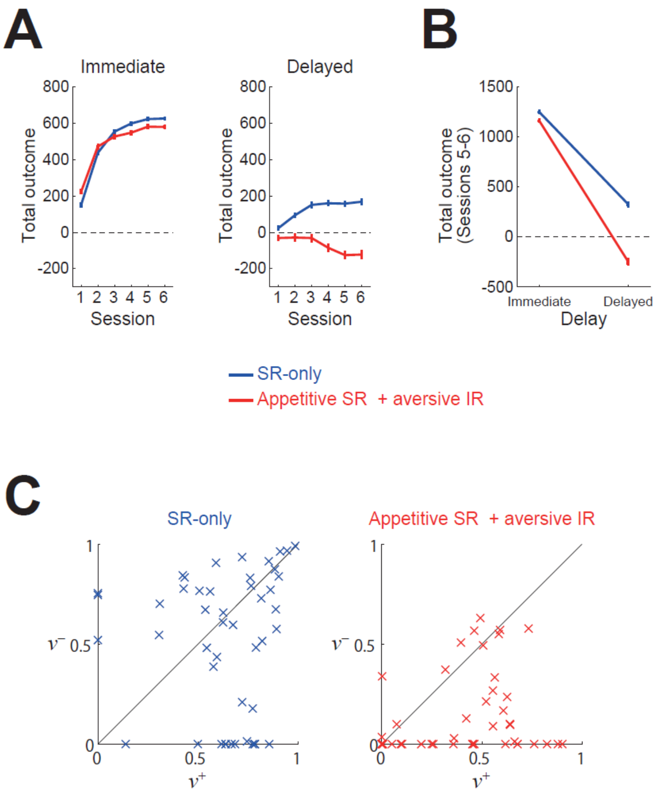

Legend

(Lines 701-711)

Behavior of the dual-system agents in the delayed feedback task examined in [10]. **(A)** Total obtained outcome (feedback) from stimuli causing immediate (left) or delayed feedback (right) in each session, averaged across 1000 simulations, for the appetitive SR + aversive IR agent (red) and the SR-only agent (blue). The error-bars indicate ±SEM. **(B)** Total obtained outcome (feedback) in sessions 5 and 6, averaged across 1000 simulations, from stimuli with immediate or delayed feedback for the two types of agents. The error-bars indicate ±SEM. **(C)** Results of fitting of the choices of the two types of agents (47 and 45 out of 50 simulations for each, separately conducted from those shown in (B)) by the separate eligibility trace actor-critic model considered in [10]. The horizontal and vertical axes indicate the estimated parameters v^+ and v^- (decay time scale of the trace for positive and negative RPEs), respectively.

Materials & Methods

(Lines 1225-1279)

Simulation and fitting of the delayed feedback task

We simulated execution of the delayed feedback task [10] by the dual-system agents. There were eight stimuli, which caused +40, +10, −10, or −40 immediate feedback (denoted as $+40^{im}$ etc) or +40, +10, −10, or −40 delayed feedback three trials later (denoted as $+40^{de}$ etc). The task consisted of six sessions, and in each session, each of six pairs of stimuli (($+10^{im}$, $+40^{im}$), ($+10^{de}$, $+40^{de}$), (-10^{im}, -40^{im}), (-10de, -40de), ($+10^{im}$, $+40^{de}$), (-10^{im}, -40de)) was presented ten times and each of ten pairs (($+40^{im}$,

$+40^{de}$), ($+10^{im}$, $+10^{de}$), (-10^{im}, -10de), (-40^{im}, -40de), ($+10^{de}$, $+40^{im}$), (-10de, -40^{im}), ($+10^{im}$, -10^{im}), ($+40^{im}$, -40^{im}), ($+10^{de}$, -10de), ($+40^{de}$, -40de)) was presented five times in a pseudorandom order (in the same manner as in the original study [10]).

The IR-based system of the dual-system agent learned the system-specific values of eight actions to choose each of the eight stimuli through updating the value of taken action by RPE multiplied by the learning rate (α_{IR+} or α_{IR-} for positive or negative RPE, respectively). The SR-based system represented each action by estimated discounted cumulative occupancies of successor "states". Seven states were defined based on the presented (total) feedback at each trial: S_1: +80, S_2: +50 or +40 or +30, S_3: +20 or +10, S_4: 0, S_5: −10 or −20, S_6: −30 or −40 or −50, and S_7: −80. At each trial k, "trace" of each action A_i (i = 1, .., 8), $T(A_i)$, was updated as

$T(A_i) \leftarrow \gamma T(A_i)$ (if A_i was not taken at trial k) or

$T(A_i) \leftarrow \gamma T(A_i) + 1$ (if A_i was taken at trial k),

where γ was the time discount factor set to 0.75. Then, a column of 8×7 SR matrix **M** that corresponded to the state at trial k (determined by the feedback presented at trial k), S_j, was updated to be equal to the traces for each action:

$\mathbf{M}_{ij} \leftarrow T(A_i)$ (i = 1, .., 8),

after **M** was used for value calculation and value (weight) update (mentioned below). The other columns of **M** remained unchanged. The SR-based system-specific value of A_i (i = 1, .., 8) was given by the dot product of the SR feature vector for A_i, i.e., i-th row of **M**, $\mathbf{M}_{(i)}$, and a weight vector **w**, i.e., $\mathbf{w} \cdot \mathbf{M}_{(i)}$. The weight **w** was updated based on the action A_i that was taken at trial k and RPE δ:

$\mathbf{w} \leftarrow \mathbf{w} + \alpha_{SR+}\delta\mathbf{M}_{(i)}$ (when $\delta \geq 0$) or

$\mathbf{w} \leftarrow \mathbf{w} + \alpha_{SR-}\delta\mathbf{M}_{(i)}$ (when $\delta < 0$).

Integrated action values were calculated as the average of the IR- and SR-based system-specific action values. Choice between two actions corresponding to two stimuli presented at each trial was made in the soft-max manner with the degree of exploitation over exploration (inverse temperature) (β) set to 0.25. RPE was calculated as the difference between the (total) feedback and the integrated value of taken action. The IR-based system-specific values for all the actions and all the elements of the weight vector for the SR-based system-specific values (**w**) decayed at a constant rate (0.001), i.e., were multiplied by 0.999 at each trial. The traces for actions $T(A_i)$, the elements of the SR matrix **M**, and the elements of the weight vector **w** were all initialized to 0. The learning rates were set as (α_{SR+}, α_{SR-}, α_{IR+}, α_{IR-}) = (0.02, 0.02, 0, 0) or (0.018, 0.002, 0.002, 0.018) for the SR-only agent or the appetitive SR + aversive IR agent, respectively. We conducted 1000 simulations for each of the two types of agents, and calculated the averages and the SEMs.

We also conducted fitting of the choices of these two types of dual-system agents (50 simulations for each, separately conducted from the abovementioned 1000 simulations) by the separate eligibility trace actor-critic model considered in [10]. This model had four free parameters: a (learning rate), b (inverse temperature for choice), and v^+ and v^- (decay time scale of the trace for positive and negative RPEs, respectively). Since a, v^+, and v^- should normally be between 0 and 1, these variables were transformed into atanh($2x$−1), where x represents a, v^+, or v^-, in the fitting process so that the range of exploration was effectively bound to [0 1]. For each choice sequence, we explored a set of parameters that maximized the log likelihood [60] by using the "fminsearch" function of MATLAB, with the initial parameter values set as (a, b) = (0.0011, 2.96), which were the medians of estimated parameters of HCs in [10], and v^+, v^- = 0.1, 0.5, or 0.9 (i.e., 3 × 3 = 9 cases were examined) and the 'MaxFunEvals' and 'MaxIter' options of this function set to both 10000. Among the nine cases with different initial values of (v^+, v^-), we selected the cases in which the "fminsearch" function terminated with "exitflag" 1 (meaning normal termination), and if there were any such case(s) (this was true for 47 and 45 simulations for the SR-only and the appetitive SR + aversive IR agent, respectively), we selected a case that gave the maximum log likelihood among them, and plotted the corresponding values of v^+ and v^- in Figure 7C.

Regarding the second question, thank you very much for letting us notice that the data of Gillan et al., 2016 has been made publicly available (we were carelessly unaware of it). We extracted the data and analyzed the choice of participants with high scores of OCI-R (≥ 40) in their Experiments 1 and 2 (23 out of 548 and 58 out of 1413 participants, respectively). The choice in Experiment 2 (Figure R2.4.2A-bottom) tended to exhibit an asymmetry in the common-vs-rare difference in the "stay" proportion between after reward and after no-reward (comparison of $(P(\text{Stay})|_{\text{common}} - P(\text{Stay})|_{\text{rare}})|_{\text{reward}}$ and $(P(\text{Stay})|_{\text{rare}} - P(\text{Stay})|_{\text{common}})|_{\text{no-reward}}$, $t(22) = -1.31$, $p = 0.203$ (Experiment 1), $t(57) = -1.99$, $p = 0.0516$ (Experiment 2)).

The direction of this asymmetry, however, is opposite from the asymmetry predicted by our appetitive SR + aversive IR agent (Figure R2.4.2C-top), which appears potentially consistent with the result for OCD patients in the reward version of the task in Voon et al., 2015 (Figure R2.4.2B-top) as we discussed in the original manuscript, but instead the same as the (apparent) asymmetry in the result for OCD patients in the punishment version in Voon et al., 2015 (Figure R2.4.2B-bottom).

We speculate as follows. In Gillan et al., 2016, "*Participants were paid a base rate (Experiment 1: $2, Experiment 2: $2.50) in addition to a bonus based on their earnings during the reinforcement-learning task (In each experiment, M=$0.54, SD=0.04)* (sentence extracted from page 13 of Gillan et al., 2016)". Given that completion of the task (200 trials) would require a considerable amount of time, these payments were rather low. So it seems possible that the participants had expected to obtain much performance-dependent bonus and they felt every failure as a punishment, even though outcomes were facially presented as reward vs no-reward rather than no-punishment vs punishment, and thus their behavior (in particular, the asymmetry in the common-vs-rare difference in the stay proportion) resembled that of OCD patients in the punishment version, rather than the reward version, of the task in Voon et al. 2015.

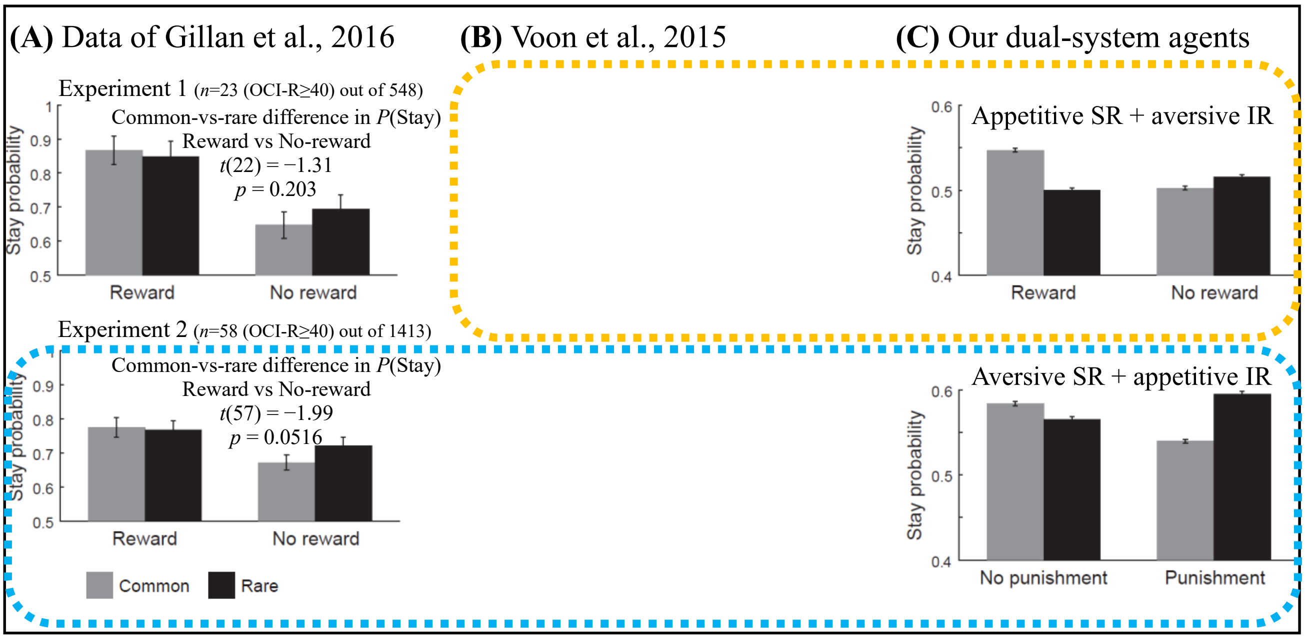

Figure R2.4.2 ((A) & (C)-bottom: Figure 9 in the manuscript; (C)-top: Figure 6B in the manuscript)

Then, the apparent contradiction in the direction of asymmetry between our model's prediction and the results of the punishment version of Voon et al. / Gillan et al. could potentially be resolved by considering the dopamine (DA) neurons representing threat/aversiveness prediction errors and basal ganglia (BG) circuit engaging in threat reinforcement learning (RL) (Matsumoto & Hikosaka, 2009, *Nature*; Menegas et al., 2018, *Nat Neurosci*; Akiti et al., 2022, *Neuron*; Ishino et al., 2023, *Sci Adv*). Specifically, it is conceivable that greater learning of SR-based system from positive than negative DA signals in OCD, suggested by our model, occurs not only in the canonical BG-DA circuit for reward RL but also in the circuit for threat/aversiveness RL, potentially through common mechanisms for projection preferences (as we had discussed in the original manuscript). Then, the latter circuit, which is recruited in punishment contexts, is expected to implement the aversive SR + appetitive IR leaner (Figure R2.4.3), which exhibits the same direction of asymmetry as observed in the punishment version of Voon et al. / Gillan et al. as we have confirmed by simulation (Figure R2.4.2C-bottom).

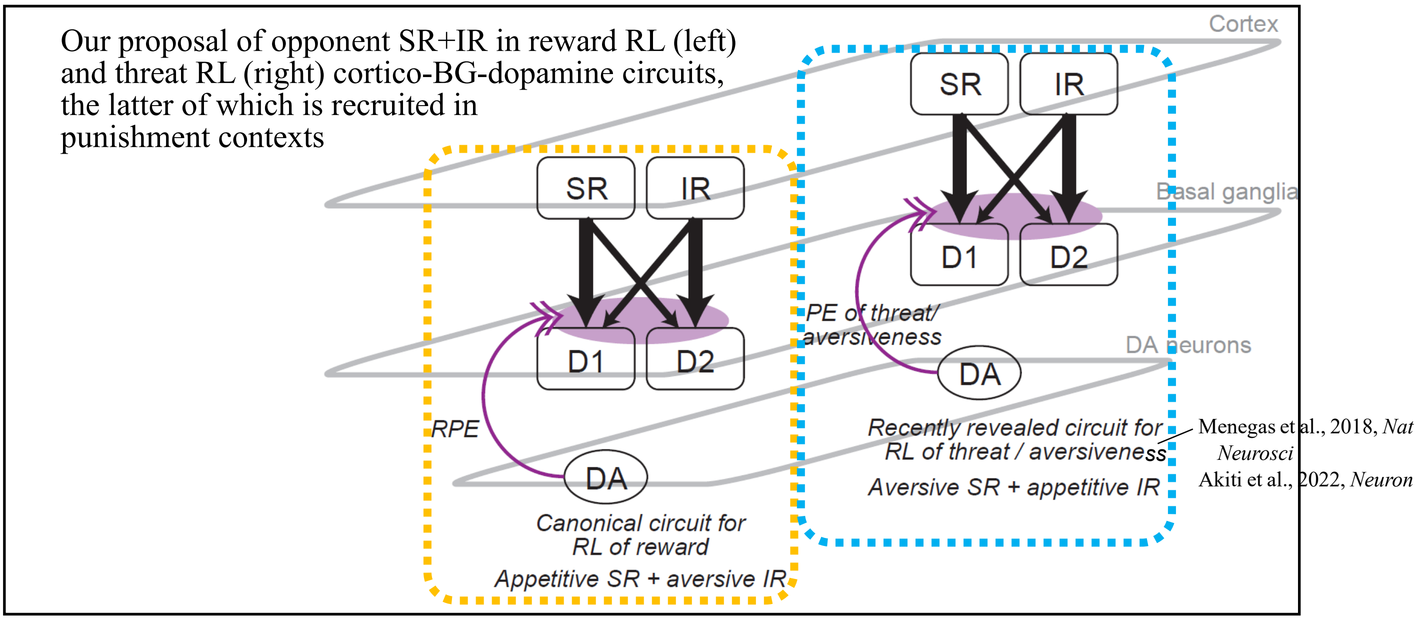

Figure R2.4.3 (Figure 8 in the manuscript)

<Descriptions/figures in the revised manuscript>

Discussion

(Lines 871-883)

However, this contradiction could potentially be resolved by considering recent findings of dopamine neuronal subpopulation representing threat prediction errors (PEs) rather than RPEs and striatal subpopulations engaged in threat (rather than reward) learning/management, including the tail of the striatum (TS) and substantia nigra pars lateralis (SNL) in mice [51-53], and also dopamine neuronal subpopulations potentially representing error signals for aversive outcome [54] or reward-omission [55]. Specifically, it is conceivable that greater learning of SR-based system from positive than negative dopamine signals in OCD suggested by our model occurs not only in the canonical cortico-basal ganglia-dopamine circuit for reward RL but also in the circuit for RL of threat or aversiveness, potentially through common mechanisms for corticostriatal projection preferences. Then, the latter circuit, which is recruited in punishment contexts, should implement the aversive SR + appetitive IR agent (Figure 8) and show (over)generalization of threat or aversiveness, potentially in line with the abovementioned conjecture. Existence of such multiple opponent SR+IR circuits could further explain the previous

mixed results regarding learning rate biases in OCD discussed above.
(Lines 944-982)

Predictions, comparisons with actual data, and future perspectives

Our model predicts that OCD patients show smaller common-vs-rare differences in the "stay" proportion after no-reward than after reward in the two-stage decision task (Figure 6B, right panel). This pattern looks similar to the actually observed pattern based on 48 OCD subjects reported in the previous study [7] (their Supplementary Figure 1), although not similar to the pattern based on 32 OCD subjects reported in their preceding study [6] (their Supplementary Figure S1). Statistical test of this specific prediction is desired to be conducted in the future.

Looking at the choice pattern of OCD subjects in the punishment version of the two-stage task (Supplementary Figure 1 of [7]), apparently, OCD subjects tended to show smaller common-vs-rare differences in the "stay" proportion after no-punishment than after punishment. If punishment/no-punishment were equated with no-reward/reward considering the sign of RPEs, the direction of this asymmetry is opposite to the direction in the reward version of the task, and thus opposite to our model's prediction. However, if greater SR-based learning from positive dopamine signals suggested by our model occurs also in the circuit for RL of threat/aversiveness, which is recruited in punishment contexts, as discussed above (Figure 8), such a direction is exactly predicted, as we confirmed by simulation of the aversive SR + appetitive IR agent (Figure 9A, bottom).

The data of the study examining the behavior in the two-stage task and self-report assessment of OCD using the Obsessive-Compulsive Inventory - Revised (OCI-R) [59] in general populations [9] is publicly available (https://osf.io/usdgt/). We extracted the data and analyzed the choice of participants with high scores of OCI-R (≥ 40) in their Experiments 1 and 2 (23 out of 548 and 58 out of 1413 participants, respectively). The choice in Experiment 2 (Figure 9B, bottom) showed a tendency of smaller common-vs-rare differences in the "stay" proportion after reward than after no-reward (comparison of $(P(\text{Stay})|_{\text{common}} - P(\text{Stay})|_{\text{rare}})|_{\text{reward}}$ and $(P(\text{Stay})|_{\text{rare}} - P(\text{Stay})|_{\text{common}})|_{\text{no-reward}}$, $t(57) = -1.99$, $p = 0.0516$ (Experiment 2); $t(22) = -1.31$, $p = 0.203$ (Experiment 1)). This direction of asymmetry is the same as the direction apparently shown by OCD subjects in the punishment version, rather than the reward version [7]. We think this could potentially be explained as follows. In the study using general populations [9], "*Participants were paid a base rate (Experiment 1: $2, Experiment 2: $2.50) in addition to a bonus based on their earnings during the reinforcement-learning task (In each experiment, M=$0.54, SD=0.04)* (sentence extracted from page 13 of [9])". Given that completion of the task (200 trials) would require a considerable amount of time, these payments were rather low. So it seems possible that the participants had expected to obtain much performance-dependent bonus and they felt every failure as a punishment, even though outcomes were facially presented as reward vs no-reward. Future study is desired to examine this possibility by manipulating the amount of payment. In terms of our model, the asymmetry in Experiment 2 can again be explained by the aversive SR + appetitive IR agent (Figure 9A, bottom).

Figure 8

(basically the same as Figure R2.4.3 in the previous page)

Legend

(Lines 908-918)

Schematic diagram of the proposed opponent SR + IR learning in multiple parallel cortico-basal ganglia (BG)-dopamine (DA) circuits. It is hypothesized that there exist preferential connections from the cortical populations having SR and IR to the striatal direct and indirect pathway neurons expressing D1 and D2 receptors, respectively. Such preferential connections cause greater learning of SR- and IR-based systems from positive and negative DA signals, respectively. It implements the appetitive SR + aversive IR agent in the canonical cortico-BG-DA circuit for reward reinforcement learning (RL), in which DA represents reward prediction error (RPE) (left part of this figure). In contrast, in the recently

revealed cortico-BG-DA circuit for threat/aversiveness RL, in which DA represents threat/aversiveness PE, the same greater learning of SR/IR-based systems from positive/negative DA signals implements the aversive SR + appetitive IR agent (right part of this figure).

Figure 9

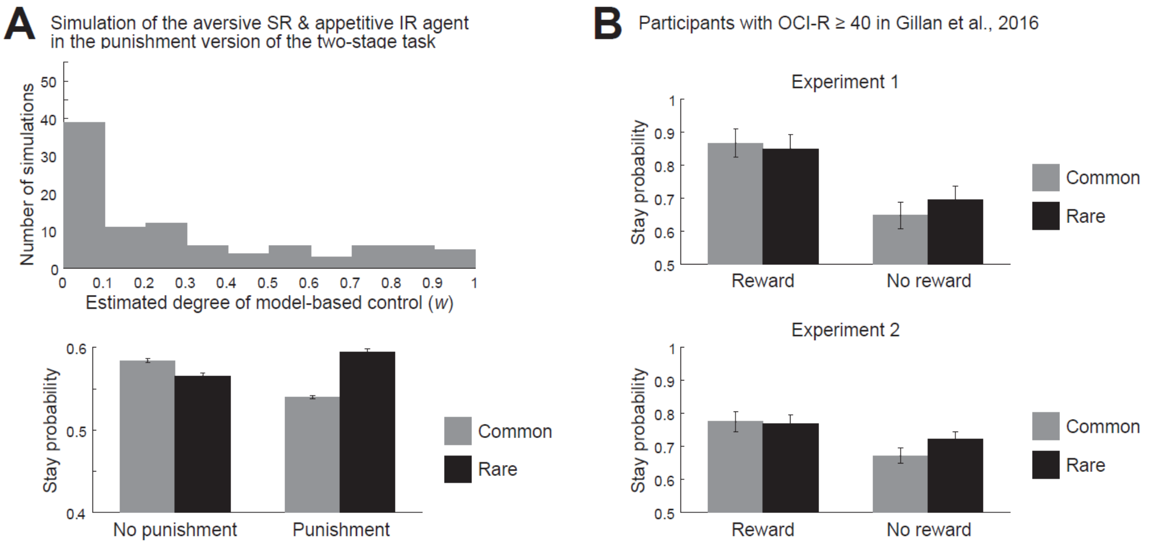

Legend

(Lines 1007-1017)

Behavior in the two-stage decision task: additional simulation result and results obtained by analysis of publicly available experimental data. **(A)** Behavior of the aversive SR + appetitive IR agent ((α_{SR+}, α_{SR-}, α_{IR+}, α_{IR-}) = (0.03, 0.27, 0.27, 0.03)) in the punishment version of the two-stage task. *Top*: Distribution of the estimated parameter representing the degree of model-based control (w) (across 98 out of 100 simulations). *Bottom*: Mean (±SEM) proportion of stay choice at the first stage across 1000 simulations. **(B)** Mean (±SEM) proportion of stay choice at the first stage of the participants with high scores of OCI-R (≥ 40) in Experiment 1 (23 out of 548 participants) (top panel) and Experiment 2 (58 out of 1413 participants) (bottom panel) of [9], obtained by analysis of the data at https://osf.io/usdgt/. Cases where the "reward" at the previous trial was negative in the data file were omitted from the analysis.

R2.5

Finally, I have to say that I found the description of the models hard to follow. The methods section could benefit from a more detailed, step-by-step exposition of the models. From a purely stylistic point-of-view – it would be much easier to read and understand the equations if they were presented on separate lines, where all components are explained.

We have revised the methods, presenting the equations on separate lines and making additions and modifications.

A few minor comments:

R2.6

・ The introduction could benefit if the relatively new 'trace imbalance model' were explained in more detail. I realize that it was developed and validated in a separate paper, but IMO, the readers should be able to understand it without having to read that other paper.

We have added more explanations in the Introduction (Lines 104-109).

R2.7

・ Figure 2 could benefit if the relevant parameters controlling the probability for abnormal reaction/compulsion are explicitly stated (and thus linked to the equations)

We have added an explanation of the actions and the probability of action selection to Figure 2.

R2.8

・ Some intuitive explanation regarding the 'weights' of the SR system mentioned in the results (and figures) can be helpful.

We have added a schematic explanation that the SR-based value is calculated as a dot product of the weights and the occupancies to the Results (Lines 395-396) and Figure 1A.

R2.9

・ On page 8, the authors say, "Now, assume that the agent, developing enhanced obsession-compulsion cycle, exists at the relief state and happens to take the "other" action repeatedly, without taking the abnormal reaction. It is in fact the optimal policy, and because no reward or punishment is given as long as the agent only takes the "other" action, the value of the "other" action should approach, through RPE-based update, to 0, which is equal to the optimal value. However, this value, 0, is lower than the positive value of the abnormal reaction (even though it slowly decays)". This is an interesting result. My main question is whether we could also predict the opposite in the case of an agent of chooses the abnormal reaction repeatedly?

If the agent repeatedly takes the abnormal reaction when the agent is at the relief state, the agent will experience frequent transitions to the obsession state, which has a low value (because it is likely to be followed by immediate punishment), and so the value of the abnormal reaction is expected to gradually decrease even in the appetitive SR + aversive IR agent (with limited learning of the abnormal reaction directly from negative RPE at the time of punishment). But then the probability of selecting the abnormal reaction (according to the soft-max choice rule) should decrease, and so eventually the

agent will switch to take the other at the relief state.

R2.10

・ The idea that the learning rate biases in the SR/IR systems are 'evolutionarily selected' is interesting, but I couldn't understand the exact proposed function of this setting. The authors cite a paper examining this in dynamic environments, but it would be helpful if they could briefly explain the logic here.

We have added an explanation to this place (Lines 731-734).

R2.11

・ In my opinion, the limitations section, and especially the second limitation – is too specific. I believe this section would benefit if it would focus on more conceptual-level limitations (e.g., what clinical/psychological phenomena the model cannot explain? To what extent are the simulations results dependent upon specific fixed parameters or settings of the model's environment?

We have added a paragraph describing limitations of our work from a more general viewpoint (Lines 923-942). Please see also our response to your point 2, which is related to this issue.

Reviewer #3:

The paper by Sato and colleagues proposes a computational reinforcement learning model of obsessive compulsive disorder symptoms. The model proposes a reinforcement learning agent that learns to predict action values from multiple representations: one over successor representations (SR) and one over independent representations (IR). The key feature of the model which the paper proposes can underlie obsessive compulsive symptoms is that learners for the SR and IR representations differentially learn from positive and negative reward prediction errors. Specifically, the SR learns more from positive reward prediction errors, and the IR learns more from negative reward prediction errors.

The claim of the paper is that this model can link together two separate findings relating to decision-making and OCD symptoms. The first is findings which that individuals with OCD have higher eligibility traces for positive compared to negative learning. The authors account for these findings by showing that, like a model with imbalanced eligibility traces, their model can also generate obsessive-compulsive cycles in a toy two-state MDP example.

The second is that individuals with OCD show less model-based learning on the two-step task. This is accounted for by showing that their model demonstrates less model-based behavior than an agent that decides just using the SR.

Overall, I think the attempt to relate compulsive symptoms to the types of representations that individuals employ in reinforcement learning is commendable and also could be fruitful approach. However, I have a number of critiques and suggestions for further work for the specific simulations presented in the paper.

R3.1

1. Simulation of Sakai et al., 2022. The first part of the paper attempts to offer a re-interpretation of previous findings from Sakai et al., 2022, which argued that individuals with OCD have a higher eligibility trace for positive compared to negative prediction errors. The current paper argues that a model where an SR learner has a higher learning rate for positive prediction errors compared to negative prediction errors, and an IR learner has the opposite pattern, can generate equivalent behavior. This is demonstrated by simulating behavior in a toy MDP and showing the model can develop obsessive-compulsive cycles that were generated in Sakai et al., 2022.

I think to make the argument though that an SR + IR learner can account for the model of Sakai et al., 2022, it is also necessary to demonstrate that the new model can generate the behavior of OCD individuals (and variation between OCD and healthy controls) from the empirical study presented in Sakai et al, 2022 which actually provides evidence for the eligibility trace account. Can an SR an IR based learner with different learning rates for positive versus negative prediction errors explain how

controls varied from individuals with OCD in the experiments that were run in Sakai et al., 2022? This should be demonstrated with simulation if the authors want to claim that their new model can capture what the previous model was suggested to capture.

Thank you very much for pointing out this important issue. We have conducted simulations of the delayed feedback task of Sakai et al., 2022, and confirmed that our dual-system-agent model could explain the experimental results of Sakai et al.. Specifically, the appetitive SR + aversive IR agent showed a specific impairment in learning of delayed feedbacks (Figure R3.1B-top), and fitting of its choices by the separate eligibility trace model of Sakai et al. resulted in a shorter trace for negative PREs (Figure R3.1B-bottom), as compared to the SR-only agent (regarding the rationale for modeling healthy controls by the SR-only agent, please see the last part of our response to your point 3 below).

(A) Experiments (Sakai et al., 2022)

(B) Our simulation (dual-system agents)

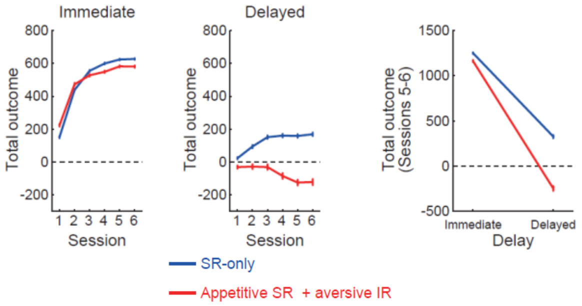

Simulation (agents with separate eligibility trace) (Sakai et al., 2022)

Fitting of the choices of dual-system agents by Sakai et al.'s separate eligibility trace model

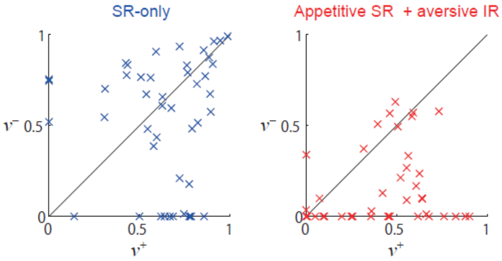

(B): Fig. 7 of manuscript

Figure R3.1

<Descriptions/figures in the revised manuscript>

Results

(Lines 639-661)

Behavior of the dual-system agents in the delayed feedback task

We examined whether the observed differential behaviors of OCD patients and HCs in a delayed feedback task [10] could be explained by our dual-system agents. In the task, a pair of stimuli were presented, and subjects were required to choose one of them. Some stimuli caused immediate positive or negative feedback (monetary gain or loss) in the same trial, while other stimuli caused delayed feedback three trials later. Compared to HCs, OCD patients showed intact learning of stimuli causing immediate feedback but impaired learning of stimuli with delayed feedback (Figure 3B,C of [10]), and this pattern was reproduced by models with balanced and imbalanced eligibility traces (Figure 3E,F of [10]). We simulated execution of this task by the dual-system agents, in which actions of choosing each stimulus were represented by estimated discounted cumulative occupancies of successor states defined based on presented feedback (see the Materials and Methods for details). As shown in Figure 7A,B, the appetitive SR + aversive IR agent (α_{SR+}, α_{SR-}, α_{IR+}, α_{IR-}) = (0.018, 0.002, 0.002, 0.018), as compared to

the SR-only agent (α_{SR+}, α_{SR-}, α_{IR+}, α_{IR-}) = (0.02, 0.02, 0, 0), showed a particular impairment in learning of delayed feedback, largely reproducing the pattern observed in the experiment [10].

We further fitted the choices of these two types of dual-system agents by the separate eligibility trace actor-critic model considered in [10]. As a result, the choices of the SR-only agent were in large part fitted by the model with large eligibility traces for both positive and negative RPEs whereas the choices of the appetitive SR + aversive IR agent were in large part fitted by the model with a larger trace for positive than negative RPEs (Figure 7C), as we expected from the similarity between the eligibility trace and SR. These results indicate that the observed behaviors of OCD patients and HCs in the delayed feedback task [10] could be explained by the appetitive SR + aversive IR agent and the SR-only agent, respectively.

Figure 7

(shown in the right)

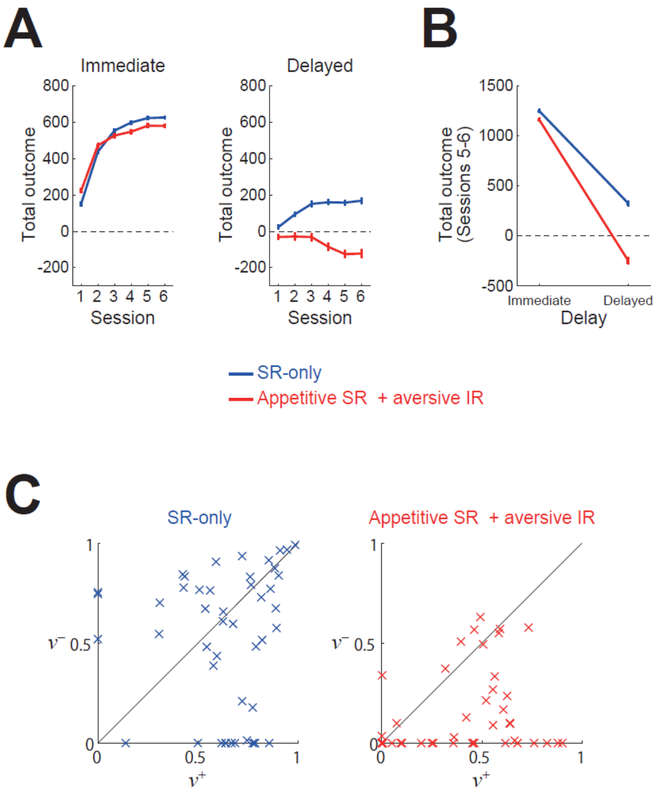

Legend

(Lines 701-711)

Behavior of the dual-system agents in the delayed feedback task examined in [10]. **(A)** Total obtained outcome (feedback) from stimuli causing immediate (left) or delayed feedback (right) in each session, averaged across 1000 simulations, for the appetitive SR + aversive IR agent (red) and the SR-only agent (blue). The error-bars indicate ±SEM. **(B)** Total obtained outcome (feedback) in sessions 5 and 6, averaged across 1000 simulations, from stimuli with immediate or delayed feedback for the two types of agents. The error-bars indicate ±SEM. **(C)** Results of fitting of the choices of the two types of agents (47 and 45 out of 50 simulations for each, separately conducted from those shown in (B)) by the separate eligibility trace actor-critic model considered in [10]. The horizontal and vertical axes indicate the estimated parameters v^+ and v^- (decay time scale of the trace for positive and negative RPEs), respectively.

Materials & Methods

(Lines 1225-1279)

Simulation and fitting of the delayed feedback task

We simulated execution of the delayed feedback task [10] by the dual-system agents. There were eight stimuli, which caused +40, +10, −10, or −40 immediate feedback (denoted as $+40^{im}$ etc) or +40, +10, −10, or −40 delayed feedback three trials later (denoted as $+40^{de}$ etc). The task consisted of six sessions, and in each session, each of six pairs of stimuli (($+10^{im}$, $+40^{im}$), ($+10^{de}$, $+40^{de}$), (-10^{im}, -40^{im}), (-10de, -40de), ($+10^{im}$, $+40^{de}$), (-10^{im}, -40de)) was presented ten times and each of ten pairs (($+40^{im}$, $+40^{de}$), ($+10^{im}$, $+10^{de}$), (-10^{im}, -10de), (-40^{im}, -40de), ($+10^{de}$, $+40^{im}$), (-10de, -40^{im}), ($+10^{im}$, -10^{im}), ($+40^{im}$, -40^{im}), ($+10^{de}$, -10de), ($+40^{de}$, -40de)) was presented five times in a pseudorandom order (in the same manner as in the original study [10]).

The IR-based system of the dual-system agent learned the system-specific values of eight actions to choose each of the eight stimuli through updating the value of taken action by RPE multiplied by the learning rate (α_{IR+} or α_{IR-} for positive or negative RPE, respectively). The SR-based system represented

each action by estimated discounted cumulative occupancies of successor "states". Seven states were defined based on the presented (total) feedback at each trial: S_1: +80, S_2: +50 or +40 or +30, S_3: +20 or +10, S_4: 0, S_5: −10 or −20, S_6: −30 or −40 or −50, and S_7: −80. At each trial k, "trace" of each action A_i (i = 1, .., 8), $T(A_i)$, was updated as

$T(A_i) \leftarrow \gamma T(A_i)$ (if A_i was not taken at trial k) or

$T(A_i) \leftarrow \gamma T(A_i) + 1$ (if A_i was taken at trial k),

where γ was the time discount factor set to 0.75. Then, a column of 8×7 SR matrix **M** that corresponded to the state at trial k (determined by the feedback presented at trial k), S_j, was updated to be equal to the traces for each action:

$\mathbf{M}_{ij} \leftarrow T(A_i)$ (i = 1, .., 8),

after **M** was used for value calculation and value (weight) update (mentioned below). The other columns of **M** remained unchanged. The SR-based system-specific value of A_i (i = 1, .., 8) was given by the dot product of the SR feature vector for A_i, i.e., i-th row of **M**, $\mathbf{M}_{(i)}$, and a weight vector **w**, i.e., $\mathbf{w}\cdot\mathbf{M}_{(i)}$. The weight **w** was updated based on the action A_i that was taken at trial k and RPE δ:

$\mathbf{w} \leftarrow \mathbf{w} + \alpha_{SR+}\delta\mathbf{M}_{(i)}$ (when $\delta \geq 0$) or

$\mathbf{w} \leftarrow \mathbf{w} + \alpha_{SR-}\delta\mathbf{M}_{(i)}$ (when $\delta < 0$).

Integrated action values were calculated as the average of the IR- and SR-based system-specific action values. Choice between two actions corresponding to two stimuli presented at each trial was made in the soft-max manner with the degree of exploitation over exploration (inverse temperature) (β) set to 0.25. RPE was calculated as the difference between the (total) feedback and the integrated value of taken action. The IR-based system-specific values for all the actions and all the elements of the weight vector for the SR-based system-specific values (**w**) decayed at a constant rate (0.001), i.e., were multiplied by 0.999 at each trial. The traces for actions $T(A_i)$, the elements of the SR matrix **M**, and the elements of the weight vector **w** were all initialized to 0. The learning rates were set as (α_{SR+}, α_{SR-}, α_{IR+}, α_{IR-}) = (0.02, 0.02, 0, 0) or (0.018, 0.002, 0.002, 0.018) for the SR-only agent or the appetitive SR + aversive IR agent, respectively. We conducted 1000 simulations for each of the two types of agents, and calculated the averages and the SEMs.

We also conducted fitting of the choices of these two types of dual-system agents (50 simulations for each, separately conducted from the abovementioned 1000 simulations) by the separate eligibility trace actor-critic model considered in [10]. This model had four free parameters: a (learning rate), b (inverse temperature for choice), and v^+ and v^- (decay time scale of the trace for positive and negative RPEs, respectively). Since a, v^+, and v^- should normally be between 0 and 1, these variables were transformed into atanh(2x−1), where x represents a, v^+, or v^-, in the fitting process so that the range of exploration was effectively bound to [0 1]. For each choice sequence, we explored a set of parameters that maximized the log likelihood [60] by using the "fminsearch" function of MATLAB, with the initial parameter values set as (a, b) = (0.0011, 2.96), which were the medians of estimated parameters of HCs in [10], and v^+, v^- = 0.1, 0.5, or 0.9 (i.e., 3 × 3 = 9 cases were examined) and the 'MaxFunEvals' and 'MaxIter' options of this function set to both 10000. Among the nine cases with different initial values of (v^+, v^-), we selected the cases in which the "fminsearch" function terminated with "exitflag" 1 (meaning normal termination), and if there were any such case(s) (this was true for 47 and 45 simulations for the SR-only and the appetitive SR + aversive IR agent, respectively), we selected a case that gave the maximum log likelihood among them, and plotted the corresponding values of v^+ and v^- in Figure 7C.

R3.2

2. Updates for SR system. I think something is going a bit wrong with how the SR learning is implemented, which is leading it to somewhat bizarre behavior in the simulation of obsession-

compulsion cycle. This is clear from the weights and value estimates that are being learned by the SR, and plotted in Fig. 4B and D. In particular, it should not be possible for the value of any action to be above 0, since there are only negative rewards in the task.

My guess is that the cause of such divergent large weights is in how weights for the SR-based system are being updated, and that the representation is not being appropriately scaled for the update. This in turn is causing learning rates that are effectively above 1. Positive prediction errors with learning rates greater than 1 can cause the SR learner to form positive value estimates even when there are no positive rewards in the task.

To explain this more technically: the equation that is used to learn weights for the SR system is w_new <- w_old + M_transpose*delta*alpha, where M is the feature vector (a row vector here) for the state from which the action was taken, delta is the reward prediction error, and alpha is the learning rate. Typically, when implementing this update, it is necessary to scale the feature vector for the update. Specifically, M needs to be divided by its dot-product with itself (M*M_transpose). This is reported in the methods section of Russek et al., 2017, PLOS Computatainal Biology, which uses this update to simulate some aspects of MB behavior. Without scaling the feature vector, the learning rate basically loses meaning, and it can wind up effectively being above one.

To explain this, consider the usual TD update, for tabular TD:

V_new <- V_old + alpha*delta,

where alpha is the learning rate and delta is the prediction error. Here, alpha has an interpretation as the amount that V changes as a proportion of the prediction error:

(V_new - V_old) = alpha*delta.

For the linear-feature function approximation, applying the same idea, we have

(V_new - V_old) = M*w_new - M*w_old.

For the update, w_new <- w_old + M*delta*alpha, if M is not scaled appropriately, the net change to V after the update is:

(V_new - V_old) = M*w_new - M*w_old
= M(w_new - w_old) -- factoring out M
= M(w_old + M_transpose*delta*alpha - w_old) -- replacing w_new with the update rule
= M*M_transpose*delta*alpha -- simplifying and multiplying in M)

So, effectively, the change in V is no longer determined by the learning rate alpha, but rather by M*M_transpose*alpha. Thus, for alpha to have the same meaning it has in tabular TD, the feature vector thus needs to be scaled in the update by M*M_transpose.

Without this scaling, when components of M are above 1 (which is what happens in tasks where states can be visited multiple times, as in the OCD example) the effective learning rate can take on values greater than 1. This can cause the SR-system's values to go above 1 in tasks where all rewards are negative. This also, I think, is what causes the weights to not converge, but rather keep growing over time.

It is possible that doing these sorts of non-scaled divergent updates might be a hypothesis for some aspects of compulsive behavior, but I think it is not the one that the authors intend to test in the paper. I'd think they should thus try to re-do the simulations with scaled updates.

Alternatively, if this scaling is not the reason for the SR weights to grow so divergently, I think it is important for the authors to discover why the SR behaves so erratically in this task. This is necessary to understand how the relevant obsessive-compulsive behavior is being produced.

As you pointed out, because of the recurrent structure of our environmental model, elements of the SR matrix became larger than 1. In our setting, because we set the time discount factor γ to 0.5, upper-bound was $1 + \gamma + \gamma^2 + ... = 2$, and M*M_transpose was upper-bounded at 2*2=4. We varied the learning rate in the range of ≤ 0.1, and so it is considered that effective learning rate was 0.4 or smaller and did not exceed 1. We have however conducted simulations with the scaling of feature vector, and confirmed that our main result (enhancement of obsession-compulsion cycle in the appetitive SR + aversive IR agent) still held (Figure R3.2.1) while the SR weight and IR value still diverged when decay of weight/value was not assumed (Figure R3.2.2). We could replace the figures to show these results, but we would prefer the original setting because it seems unclear whether the scaling of feature vector at every time step can be biologically implemented and biological validity is one of features of our model. We have instead added a note on this issue in the Materials & Methods (Lines 1111-1118).

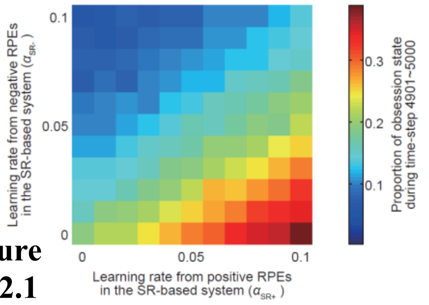

Figure R3.2.1

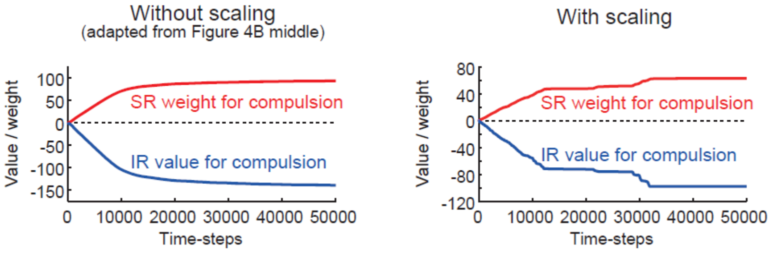

Figure R3.2.2

The reason for the SR weight and the IR value to grow divergently is considered to be in the opponent configuration. We have newly examined an agent consisting of two IR-based systems (one appetitive and one aversive), and confirmed that even though this agent did not develop enhanced obsession-compulsion cycle (Figure R3.2.3-top), the appetitive IR-based system-specific value and the aversive IR-based system-specific value of "compulsion" diverged to positive and negative directions, respectively (Figure R3.2.3-bottom). Previous opponent learning basal-ganglia models (Collins & Frank, 2014, *Psychol Rev*; Mikhael & Bogacz, 2016, *PLoS Comput Biol*), which did not have SR but only had IR, also incorporated decay/forgetting.

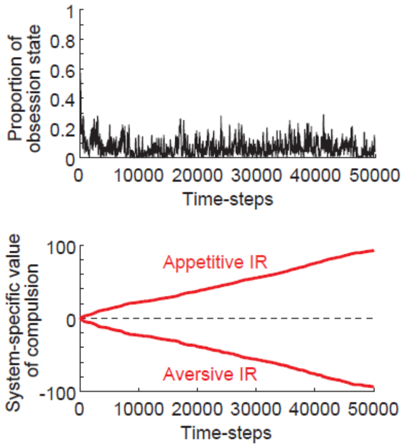

Figure R3.2.3
(Figure 4C in the manuscript)

<Descriptions/figures in the revised manuscript>

Results

(Lines 397-400)

This value/weight growth is considered to be due to the opponent setting rather than factors specific to SR, as value growth also occurred in the agent consisting of an appetitive IR-based system and an aversive IR-based system, for which obsession-compulsion cycle was not enhanced (Figure 4C).

Figure 4C

(the same as Figure R3.2.3 in the previous page)

Legend

(Lines 464-467)

Behavior of the appetitive IR ((α_{IR+}, α_{IR-}) = (0.09, 0.01)) + aversive IR ((α_{IR+}, α_{IR-}) = (0.01, 0.09)) agent. The moving average of the proportion that the agent was at the obsession state (top) and the system-specific values of the compulsion (bottom) in a single simulation are shown.

Materials & Methods

(Lines 1111-1118)

Notably, the abovementioned algorithm for TD learning of the SR-based system differed from the one described in [13], not only in the specific assumptions on the learning rates and the decay but also in that our algorithm lacked a scaling of the SR feature vector by its squared norm upon updating the weight. Without this scaling, the learning rate for weight update was effectively multiplied with the squired norm of the feature vector. Elements of the feature vectors were upper-bounded by $1 + \gamma + \gamma^2 + ... = 2$ (as we set $\gamma = 0.5$) and so the squared norm of the feature vector was upper-bounded by $2^2 = 4$. Since we varied the learning rates for weight update in the range of ≤ 0.1, effective learning rates did not exceed 1.

(Lines 1119-1123)

In order to examine the reason for the value growth occurred in the original appetitive SR + aversive IR agent, we also examined the agent consisting of an appetitive IR-based system ((α_{IR+}, α_{IR-}) = (0.09, 0.01)) and an aversive IR-based system ((α_{IR+}, α_{IR-}) = (0.01, 0.09)). As in the case of SR + IR agent, the average of the two IR-based system-specific values, initialized to 0, was used for action selection and TD-RPE calculation.

R3.3

3. Two-step task simulation. For the two-step task simulation, the authors compare their proposed model of compulsion (SR + IR with asymmetric updating) to a model which only uses the SR. They find that SR + IR with asymmetric updating produces less model-based behavior than SR alone.

This finding however is not surprising, and does not provide evidence that the proposed model (SR + IR with asymmetric updating) produces OCD behavioral patterns in the two-step task. Applied to the two-step task, the SR functions like a model-based learner and the IR functions like a model-free learner. The simulations thus effectively compare a system which combines MB (here SR) and MF (here IR) to a system that only uses MF (IR). The simulations thus don't test the need for asymmetric updating in producing OCD-like behavior (less MB). To make the argument that the proposed model generates observed OCD behavior, the authors need to compare SR and IR with asymmetric updating to SR and IR with neutral updating, and see whether this produces less MB

behavior, in a manner consistent with findings on behavior from OCD individuals. This is needed to validate the claim that asymmetric updating to SR vs IR systems generates compulsive symptoms.

We admit that it was not quite surprising that the estimated model-based weight parameter (*w*) for the opponent SR+IR agent was smaller than the estimated w for the SR-only agent, and in fact we had expected it as we described in the Introduction. However, even though a reduction of estimated *w* was expected, it was not possible to expect how much reduction occurred (in particular, whether it could be comparable to the observed reduction in OCD patients). We think our result that the opponent SR+IR agent was fitted with rather small values of *w* (Figure R3.3-middle), despite being half made of SR-based learner, is still meaningful in this regard.

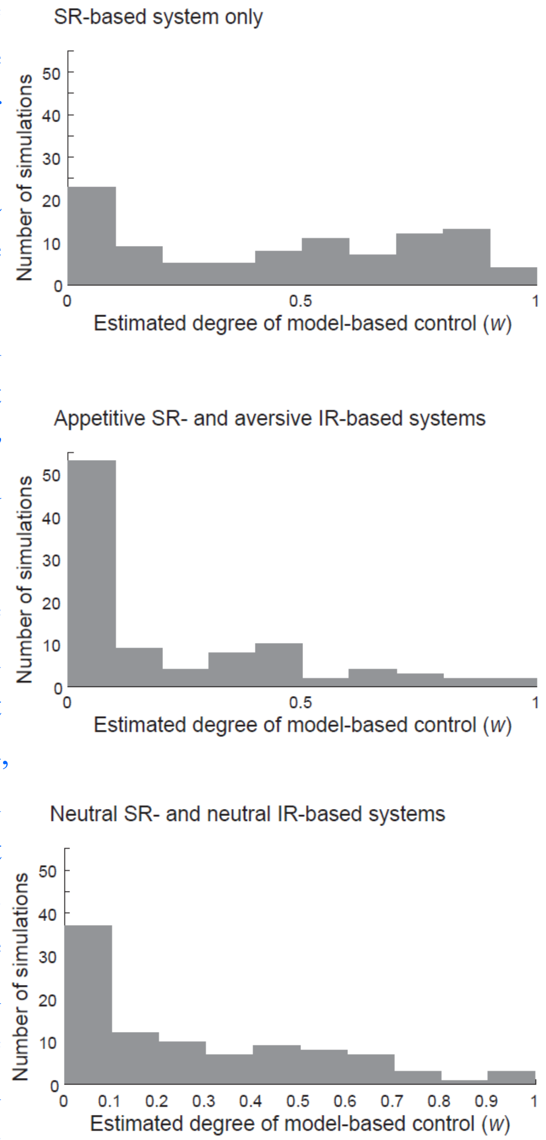

Figure R3.3

(Figure 6A,B,E in the manuscript)

However, as you pointed out, this result does not prove that the low estimated *w* in OCD patients in fact comes from such an opponent SR+IR configuration. We had not conducted estimation of *w* for the neutral SR+IR agent (α_{SR+}, α_{SR-}, α_{IR+}, α_{IR-}) = (0.15, 0.15, 0.15, 0.15) upon original submission, but we have now conducted it and found that the neutral SR+IR agent was also fitted with small values of *w* that did not drastically differ from those for the opponent SR+IR agent (Figure R3.3-bottom). Thus, from the estimation of *w* only, we cannot say which of the opponent SR+IR or the neutral SR+IR can be a better model for OCD. One support for the opponent SR+IR over the neutral SR+IR is our simulation result that only the former developed enhanced obsession-compulsion cycle (and because of this, we originally only estimated *w* for the opponent SR+IR). Empirically, the opponent SR+IR can be distinguished from the neutral SR+IR in the two-stage task by examining if there exists an asymmetry in the common-vs-rare difference in the proportion of "stay" between after reward and after no-reward (as for this issue, please see also our response to your point 4 below).

Notably, the abovementioned result that the neutral SR+IR agent was fitted by rather low values of *w* (while the SR-only agent was fitted by relatively large values of *w*) gives a rationale for modeling healthy subjects by the SR-only agent rather than by the neutral SR+IR agent also in our new

simulation of the delayed feedback task of Sakai et al. in response to your point 1 above. We consider, however, that healthy people would not always use SR(model)-based learning but may also use IR-based learning, or even appetitive SR- and aversive IR-based learning as it was shown to be advantageous in certain dynamic environments (Morita et al., 2023, *eNeuro*). Healthy people might be able to strongly rely on SR(model)-based system when appropriate while OCD patients might have an impairment in such an arbitration of behavioral controls, potentially in line with a previous suggestion of a weakness of the arbitration mechanism in OCD (Gruner et al., 2016, *Neuroscientist*).

<Descriptions/figures in the revised manuscript>

Results

(Lines 552-554)

This reduction of w was rather prominent, even though the appetitive SR + aversive IR agent was half made of SR learner, and we think it could thus potentially explain the reduction of w observed in OCD patients [7].

(Lines 564-570)

We further conducted the fitting analysis for the neutral SR + IR agent. As shown in the left panel of Figure 6E, the estimated w was typically low, and its distribution did not drastically differ from the case of the appetitive SR + aversive IR agent, although there was a tendency (Wilcoxon rank sum test, p = 0.05857). Thus, from the estimation of the weight of model-based control (w) only, we cannot say that the appetitive SR + aversive IR agent is a better model of OCD patients than the neutral SR + IR agent, while such a claim was supported by the abovementioned simulation results (Figure 3 and 4H).

(Lines 600-614)

Therefore, whether the appetitive SR + aversive IR agent or the neutral SR+IR agent better describes OCD could be distinguished by examining whether there exists such an asymmetry between after reward and after no-reward in the common-vs-rare difference in the "stay" proportion.

Discussion

(Lines 809-817)

We modeled the behavior of healthy subjects in the two-stage task by the SR-only agent rather than by the neutral SR+IR agent. This could be justified by the results that the choices of the neutral SR+IR agent were fitted with rather low weights of model-based control (w) while those of the SR-only agent were fitted by relatively large values of w (Figure 6A,E, left panels). We consider, however, that healthy people would not always use SR(model)-based learning but may also use IR-based learning, or even appetitive SR- and aversive IR-based learning when it is advantageous. Healthy people might be able to strongly rely on SR(model)-based system when appropriate while OCD patients might have an impairment in such an arbitration of behavioral control, potentially in line with a previous suggestion [44].

Figure 6E

(the same as the bottom panel of Figure R3.3 in the previous page)

Legend

(Lines 635-637)

Results for the neutral SR + IR agent ((α_{SR+}, α_{SR-}, α_{IR+}, α_{IR-}) = (0.15, 0.15, 0.15, 0.15)). Configurations are the same those in (A-D), with the left panel showing the distribution of w across 97 out of 100 simulations.

R3.4

4. Possible additional empirical analysis. This is a suggestion that the authors do not need to complete to make a publishable paper, but I think would make the paper much stronger, so they might consider. The two-step task simulations have clear predictions of what compulsive behavior in the two-step task should look like (more MB behavior following rewards compared to non-reward). This prediction could be tested in existing public datasets of the two-step task. Specifically, data from Gillan et al., 2016, which analyzed how two-step behavior varies with self-reported compulsivity symptoms is publicly available at https://osf.io/usdgt/. It should be possible to re-analyze this data and determine whether OCD symptoms are associated with more asymmetric updating of an IR + SR learner (or alternatively whether there is more MB behavior following reward compared to non-reward events in individuals higher in self-reported OCD symptoms). Such an empirical result would make the argument in the paper much stronger.

Thank you very much for letting us notice that the data of Gillan et al., 2016 has been made publicly available (we were carelessly unaware of it). We extracted the data and analyzed the choice of participants with high scores of OCI-R (≥ 40) in their Experiments 1 and 2 (23 out of 548 and 58 out of 1413 participants, respectively). The choice in Experiment 2 (Figure R3.4.1A-bottom) tended to exhibit an asymmetry in the common-vs-rare difference in the "stay" proportion between after reward and after no-reward (comparison of $(P(\mathrm{Stay})|_{\mathrm{common}} - P(\mathrm{Stay})|_{\mathrm{rare}})|_{\mathrm{reward}}$ and $(P(\mathrm{Stay})|_{\mathrm{rare}} - P(\mathrm{Stay})|_{\mathrm{common}})|_{\mathrm{no\text{-}reward}}$, $t(22) = -1.31$, $p = 0.203$ (Experiment 1), $t(57) = -1.99$, $p = 0.0516$ (Experiment 2)).

The direction of this asymmetry, however, is opposite from the asymmetry predicted by our appetitive SR + aversive IR agent (Figure R3.4.1C-top), which appears potentially consistent with the result for OCD patients in the reward version of the task in Voon et al., 2015 (Figure R3.4.1B-top) as we discussed in the original manuscript, but instead the same as the (apparent) asymmetry in the result for OCD patients in the punishment version in Voon et al., 2015 (Figure R3.4.1B-bottom).

We speculate as follows. In Gillan et al., 2016, "*Participants were paid a base rate (Experiment 1: \$2, Experiment 2: \$2.50) in addition to a bonus based on their earnings during the reinforcement-learning task (In each experiment, M=\$0.54, SD=0.04)* (sentence extracted from page 13 of Gillan et al., 2016)". Given that completion of the task (200 trials) would require a considerable amount of time, these payments were rather low. So it seems possible that the participants had expected to obtain much performance-dependent bonus and they felt every failure as a punishment, even though outcomes were facially presented as reward vs no-reward rather than no-punishment vs punishment, and thus their behavior (in particular, the asymmetry in the common-vs-rare difference in the stay proportion) resembled that of OCD patients in the punishment version, rather than the reward version, of the task in oon et al. 2015.

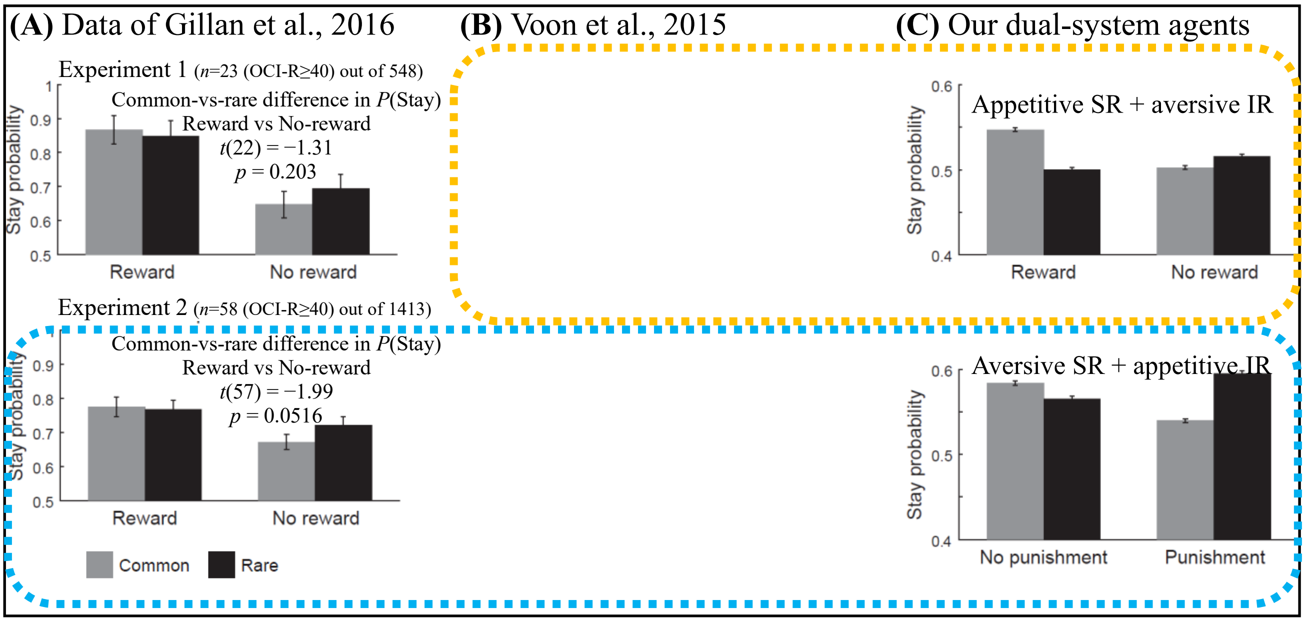

Figure R3.4.1 ((A) & (C)-bottom: Figure 9 in the manuscript; (C)-top: Figure 6B in the manuscript)

Then, the apparent contradiction in the direction of asymmetry between our model's prediction and the results of the punishment version of Voon et al. / Gillan et al. could potentially be resolved by considering the dopamine (DA) neurons representing threat/aversiveness prediction errors and basal ganglia (BG) circuit engaging in threat reinforcement learning (RL) (Matsumoto & Hikosaka, 2009, *Nature*; Menegas et al., 2018, *Nat Neurosci*; Akiti et al., 2022, *Neuron*; Ishino et al., 2023, *Sci Adv*). Specifically, it is conceivable that greater learning of SR-based system from positive than negative DA signals in OCD, suggested by our model, occurs not only in the canonical BG-DA circuit for reward RL but also in the circuit for threat/aversiveness RL, potentially through common mechanisms for projection preferences (as we had discussed in the original manuscript). Then, the latter circuit, which is recruited in punishment contexts, is expected to implement the aversive SR + appetitive IR leaner (Figure R3.4.2), which exhibits the same direction of asymmetry as observed in the punishment version of Voon et al. / Gillan et al. as we have confirmed by simulation (Figure R3.4.1C-bottom).

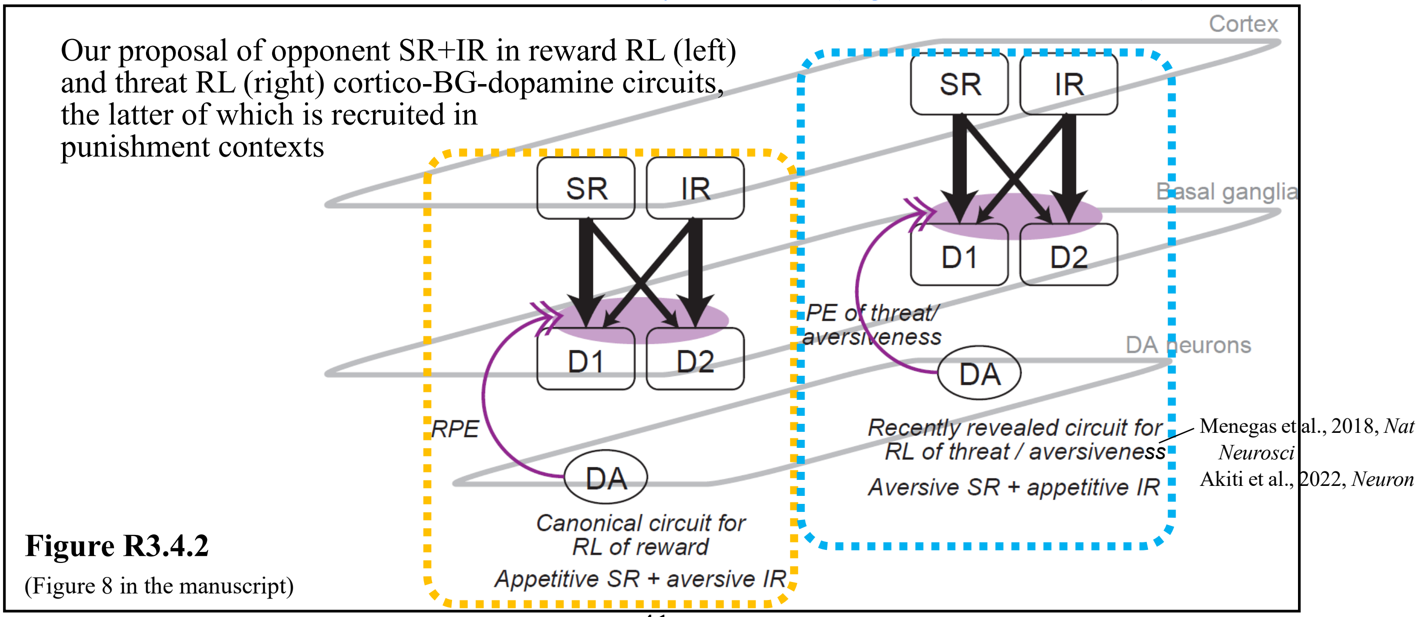

Figure R3.4.2

(Figure 8 in the manuscript)

<Descriptions/figures in the revised manuscript>

Discussion

(Lines 871-883)

However, this contradiction could potentially be resolved by considering recent findings of dopamine neuronal subpopulation representing threat prediction errors (PEs) rather than RPEs and striatal subpopulations engaged in threat (rather than reward) learning/management, including the tail of the striatum (TS) and substantia nigra pars lateralis (SNL) in mice [51-53], and also dopamine neuronal subpopulations potentially representing error signals for aversive outcome [54] or reward-omission [55]. Specifically, it is conceivable that greater learning of SR-based system from positive than negative dopamine signals in OCD suggested by our model occurs not only in the canonical cortico-basal ganglia-dopamine circuit for reward RL but also in the circuit for RL of threat or aversiveness, potentially through common mechanisms for corticostriatal projection preferences. Then, the latter circuit, which is recruited in punishment contexts, should implement the aversive SR + appetitive IR agent (Figure 8) and show (over)generalization of threat or aversiveness, potentially in line with the abovementioned conjecture. Existence of such multiple opponent SR+IR circuits could further explain the previous mixed results regarding learning rate biases in OCD discussed above.

(Lines 944-982)

Predictions, comparisons with actual data, and future perspectives

Our model predicts that OCD patients show smaller common-vs-rare differences in the "stay" proportion after no-reward than after reward in the two-stage decision task (Figure 6B, right panel). This pattern looks similar to the actually observed pattern based on 48 OCD subjects reported in the previous study [7] (their Supplementary Figure 1), although not similar to the pattern based on 32 OCD subjects reported in their preceding study [6] (their Supplementary Figure S1). Statistical test of this specific prediction is desired to be conducted in the future.

Looking at the choice pattern of OCD subjects in the punishment version of the two-stage task (Supplementary Figure 1 of [7]), apparently, OCD subjects tended to show smaller common-vs-rare differences in the "stay" proportion after no-punishment than after punishment. If punishment/no-punishment were equated with no-reward/reward considering the sign of RPEs, the direction of this asymmetry is opposite to the direction in the reward version of the task, and thus opposite to our model's prediction. However, if greater SR-based learning from positive dopamine signals suggested by our model occurs also in the circuit for RL of threat/aversiveness, which is recruited in punishment contexts, as discussed above (Figure 8), such a direction is exactly predicted, as we confirmed by simulation of the aversive SR + appetitive IR agent (Figure 9A, bottom).

The data of the study examining the behavior in the two-stage task and self-report assessment of OCD using the Obsessive-Compulsive Inventory - Revised (OCI-R) [59] in general populations [9] is publicly available (https://osf.io/usdgt/). We extracted the data and analyzed the choice of participants with high scores of OCI-R (≥ 40) in their Experiments 1 and 2 (23 out of 548 and 58 out of 1413 participants, respectively). The choice in Experiment 2 (Figure 9B, bottom) showed a tendency of smaller common-vs-rare differences in the "stay" proportion after reward than after no-reward (comparison of $(P(\text{Stay})|_{\text{common}} - P(\text{Stay})|_{\text{rare}})|_{\text{reward}}$ and $(P(\text{Stay})|_{\text{rare}} - P(\text{Stay})|_{\text{common}})|_{\text{no-reward}}$, $t(57) = -1.99$, $p = 0.0516$ (Experiment 2); $t(22) = -1.31$, $p = 0.203$ (Experiment 1)). This direction of asymmetry is the same as the direction apparently shown by OCD subjects in the punishment version, rather than the reward version [7]. We think this could potentially be explained as follows. In the study using general populations [9], "*Participants were paid a base rate (Experiment 1: $2, Experiment 2: $2.50) in addition to a bonus based on their earnings during the reinforcement-learning task (In each experiment, M=$0.54, SD=0.04)* (sentence extracted from page 13 of [9])". Given that completion of the task (200 trials) would require a considerable amount of time, these payments were rather low. So it seems possible that the participants had expected to obtain much performance-dependent bonus and they felt every failure as a punishment, even though outcomes were facially presented as reward vs no-reward.

Future study is desired to examine this possibility by manipulating the amount of payment. In terms of our model, the asymmetry in Experiment 2 can again be explained by the aversive SR + appetitive IR agent (Figure 9A, bottom).

Figure 8

(basically the same as Figure R3.4.2 (two pages before))

Legend

(Lines 908-918)

Schematic diagram of the proposed opponent SR + IR learning in multiple parallel cortico-basal ganglia (BG)-dopamine (DA) circuits. It is hypothesized that there exist preferential connections from the cortical populations having SR and IR to the striatal direct and indirect pathway neurons expressing D1 and D2 receptors, respectively. Such preferential connections cause greater learning of SR- and IR-based systems from positive and negative DA signals, respectively. It implements the appetitive SR + aversive IR agent in the canonical cortico-BG-DA circuit for reward reinforcement learning (RL), in which DA represents reward prediction error (RPE) (left part of this figure). In contrast, in the recently revealed cortico-BG-DA circuit for threat/aversiveness RL, in which DA represents threat/aversiveness PE, the same greater learning of SR/IR-based systems from positive/negative DA signals implements the aversive SR + appetitive IR agent (right part of this figure).

Figure 9

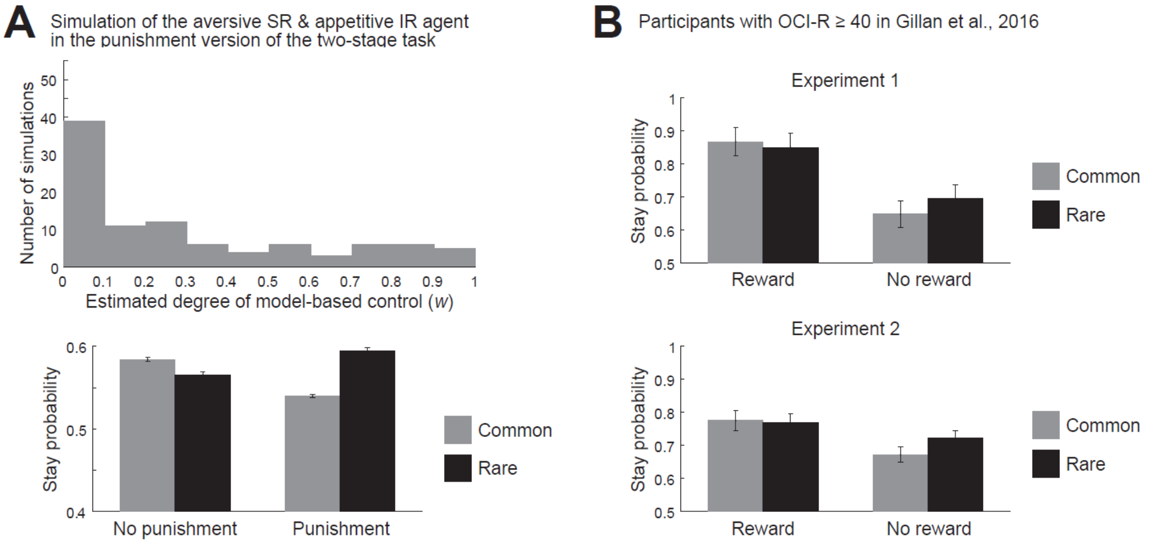

Legend

(Lines 1007-1017)

Behavior in the two-stage decision task: additional simulation result and results obtained by analysis of publicly available experimental data. **(A)** Behavior of the aversive SR + appetitive IR agent ((α_{SR+}, α_{SR-}, α_{IR+}, α_{IR-}) = (0.03, 0.27, 0.27, 0.03)) in the punishment version of the two-stage task. *Top*: Distribution of the estimated parameter representing the degree of model-based control (w) (across 98 out of 100 simulations). *Bottom*: Mean (±SEM) proportion of stay choice at the first stage across 1000 simulations. **(B)** Mean (±SEM) proportion of stay choice at the first stage of the participants with high scores of OCI-R (≥ 40) in Experiment 1 (23 out of 548 participants) (top panel) and Experiment 2 (58 out of 1413 participants) (bottom panel) of [9], obtained by analysis of the data at https://osf.io/usdgt/. Cases where the "reward" at the previous trial was negative in the data file were omitted from the analysis.
