# Supplementary material for: Opponent learning with different representations in the cortico-basal ganglia pathways can develop obsession-compulsion cycle
Source: PLoS Comput Biol. 2023 Jun 15;19(6):e1011206. doi: 10.1371/journal.pcbi.1011206 (PMC10306209; doi:10.1371/journal.pcbi.1011206)

Response to the review comments

We thank the reviewers very much for giving us the important comments. Here we attach our responses. The review comments are in black, and our responses are in blue.

Contents

- Response to Editors' comments : page 2
- Response to Reviewer #1's comments : page 3
- Response to Reviewer #2's comments : page 4-
- Response to Reviewer #3's comments : page 6-
- Copy of the newly added "An alternative environmental model" section with Figure 10 : page 9~
- Explanation of our fitting analysis of the choice data of Gillan et al.: page 12~

Notes

- The **line numbers mentioned in this document correspond to those in the "Revised manuscript with Changes Highlighted"** rather than those in the clean "Manuscript" file.
- The main modifications are summarized in the cover letter.

Editors' comments

While all the reviewers appreciated your efforts and clarifications, two reviewers (1 and 3) still present significant and reasonable doubts concerning the potential impact and solidity of your arguments. So, unfortunately, at this stage we cannot accept and the paper and we have to ask to further revise the paper while taking into account the remaining points of the reviewers (especially 1 and 3). We apologize for the quite long delay and the additional load of work that it will require, but in line with PLoS Computational Biology standard and in order to make sure that the paper delivers its promises. We specifically stress the fact that

1) to which extent the features of the models contribute to the model behavior and specific pattern (OCD cycles)

2) the stability of the results as a function of the choice of the parameters

Thank you very much for summarizing the review comments and guiding our revision. We have addressed these two points by reexamining the environmental model and exploring the parameter dependence of the agent behavior, as summarized in "1) Contribution of model features to the specific pattern of OC cycle, and parameter dependence" in the cover letter and detailed in this rebuttal letter.

Reviewer #1:

I thank the authors for their comprehensive revision - the introduction set the context and background with clear motivations and hypotheses, and the results were also signposted and explained well. I have no further questions on the points that were raised previously, only minor question/suggestions:

1. The addition of the inverse temperature analysis is quite interesting. I think there should be a little further elaboration of the role/effect of the temperature parameter in this context - does this mean individual differences in exploration/exploitation could predict whether the agent (and presumably, a human) would go into the obsessional state/develop OCD?

Thank you very much for your appreciation of our revision work, and also for this comment. We agree that the dependence on the inverse temperature potentially indicates that the individual's degree of exploration/exploitation affects vulnerability to OCD in humans. It is tempting to discuss it, but we have refrained from it because it seems unclear how (to what extent) the inverse temperature of our simple model corresponds to actual human's exploration/exploitation.

2. In the analysis for the choice patterns of the agents (Figure 6), I wonder if it would be helpful to report some statistics either with the P(Stay) difference or the reward*trans effect from the LMM (i.e., Stay ~ Reward * Transition + (Reward * Transition + 1 | Subject)), as the stay probability graphs differences are quite small.

We have compared the P(Stay) difference by paired t-test, and obtained a significant difference. We have added the following to Line 514-515:

(comparison of $(P(\text{Stay})|_{\text{common}} - P(\text{Stay})|_{\text{rare}})|_{\text{reward}}$ and $(P(\text{Stay})|_{\text{rare}} - P(\text{Stay})|_{\text{common}})|_{\text{no-reward}}$, $t(999) = 6.97$, $p = 5.93\times10^{-12}$)

Reviewer #2:

I appreciate the authors' considerable efforts in revising the manuscript, particularly the additional analyses examining the questions the other reviewers and I raised. Indeed, the additional analyses and revisions clarify and improve the manuscript.

However, these important clarifications and analyses also emphasize some considerable limitations of the proposed model, specifically with regards to it conceptual and empirical foundations.

From a conceptual point of view, the key 'psychological' mechanism through which the model explains increased obsessions seems to rely on a somewhat peculiar logic. As the reviewers now clarify, the model explains excessive 'entering into an obsessional state' as driven by the overgeneralization of the pleasantness of relief from obsession'. Such motivation for obsessions seems strange, and can be equated, in a different context, to a model that chooses to 'put its hand in the fire because it 'overgeneralizes the pleasantness of eventually taking it out of the fire'.

Furthermore, as the authors now clarify, this behavior seems to depend on several seemingly arbitrary settings of the simulations. Specifically, the model's choice to enter the obsessive state depends on the lack of enough alternative actions, and sufficient stochasticity (i.e., temperature). Along the same lines, I would assume that if the value of the other options would increase (i.e. if the model had additional, *rewarding enough* actions to choose from), this would also eliminate the obsessive behavior of the model. So, in other words, this model in the illustrative 'fire' context would 'randomly decides to put its hand in the fire because it doesn't have enough rewarding (interesting?) alternatives, and because the suffering entailed by this behavior is overweighed by the relief of eventually taking the hand out of the fire'.

Of course, the fact that this logic appears peculiar to me is not conclusive evidence against it. However, I believe the authors also do not provide sufficient empirical evidence for the model. Yes, the model can explain the results of the delayed feedback task, but so can the original 'eligibility trace' model. Yes, the model predicts reduced model-based behavior, but so does a 'neutral' SR-IR model (as correctly raised by Reviewer 3, and agreed to by the authors), and as it seems from Figure 9, an aversive SR + appetitive IR model. This latter model also appears to explain better the data from Voon (ref 7 in the manuscript), and after some speculations, the Gillan data (ref 9 in the manuscript). But, while this reversed model can, it seems, explain empirical data (as a side note, I think fitting the model to the Gillan data will be a much stronger proof here), whether it will predict obsessive behavior in the environment the authors use is questionable, and to the very least – should be examined.

Thank you very much for your critical but important comments. Reading your comment and the other reviewer's comment, we have now come to think that the explanation of obsession-compulsion

by the appetitive SR + aversive IR agent in the environmental model that we used (developed by Sakai et al.) would indeed has a significant difficulty (even though it could be argued that behavior of patients of psychiatric disorders could potentially appear to be peculiar/irrational from the normative viewpoints of healthy people). We have then realized that the aversive SR + appetitive IR agent, rather than the appetitive SR + aversive IR agent, could instead specifically develop obsession-compulsion if the environment is modeled in a (rather slightly) different manner. We have described this in the new section of the Discussion ("An alternative environmental model"), which is copied in page 9~ of this document.

We think that this alternative explanation could resolve the issues raised by you and the other reviewers (in the current and previous reviews). According to your illustrative 'fire' context, this alternative explanation corresponds to "hesitating to take one's hand out of the fire because it overgeneralizes the aversiveness of eventually putting it in the fire again". This is also irrational, but as we argued above, the irrationality itself would not generally dismiss models for psychiatric disorders, and we think that this might actually fit the characteristics of irrationally motivated compulsion. Also, as for the lack of alternative actions in the model that you pointed out, it could correspond to a situation where a person concentrates on the emerging intrusive thoughts so much that s/he cannot think of a variety of other things as we discussed in Line 667-669.

Regarding the fitting of the choice data of Gillan et al. 2016 by our dual-system agent model, we have tried, but found it technically difficult. We summarized what we have done in "2) Fitting of choice data of Gillan et al. by our dual-system model" in the cover letter, and detailed it in page 12~ of this document.

Reviewer #3:

I commend the authors for having done substantial work in their revision. In regard to my specific critiques, I think the simulation of Sakoi et al., is mostly compelling. I also think the authors have addressed my concern about why the SR weights diverge. However, my concerns over the two-step task simulations have increased.

The key claim of the paper is that OCD symptoms could be generated by a model combining SR and IR with asymmetric learning rates for positive and negative prediction errors, where SR learns more from appetitive prediction errors and IR learns more from aversive prediction errors. I commend the authors for looking at the Gilan et al., 2016 data to see to what extent this model is supported in two-step task data. I have some uncertainty about the test that was used to look for evidence that SR and IR have different learning rates for positive and negative prediction errors. I think a more straightforward approach would be to fit the model to the task data, to treat the learning rate for either system as free parameters, and then analyze how those learning rates change as a function of self-reported OCD symptoms. This could potentially also support the suggestion that healthy participants are described by SR alone (through model comparison). I found this surprising, since SR is typically thought to stand in for the model-based system, but not the model-free system, and healthy participants in the task are described by a mixture of model-based and model-free systems. I think the ability of the SR in simulations here to generate mixtures over MB and MF weightings might be due to that it learns the transitions - so this would be similar to the MB system learning the transitions from experience. This is a reasonable hypothesis for what generates behavior that might look like model-free learning (low w), and this this could potentially be supported by actual model-fitting.

Thank you very much for your comments. Regarding why the estimated weight of model-based control ("w") for our SR-only agent was not really close to 1, we did not well consider it before, and we thank you for your insight. We agree that it may reflect the fact that our SR-based system incorporates prediction error-based updates. We have added a note on this in Line 488-491:

> Notably, the distribution of the estimated *w* for SR-only agent was not very close to 1 but rather wide, and this might reflect the fact that our SR-based system incorporated PE-based updates rather than direct value calculation by multiplication of fixed SR features and the reward vector.

Regarding the fitting of the choice data of Gillan et al. 2016 by our dual-system agent model, we have tried, but found it technically difficult, presumably because of a reason related to the abovementioned point (our SR-based system incorporates prediction error-based updates). We summarized what we have done in "2) Fitting of choice data of Gillan et al. by our dual-system model" in the cover letter, and detailed it in page 12~ of this document.

More generally though, if the test used currently is correctly identifying learning rates for either system,

it falsifies the model's predictions, instead showing that MB (or SR) behavior in OCD individuals is more influenced by negative than positive prediction errors relative to MF (or IR) behavior. The paper suggests an explanation for this. First, it argues that the Gilan et al. experiment, due to low pay, should be considered to be in the punishment rather than reward domain. Second, it argues that the punishment domain might encourage opposite learning rate asymmetries - where SR would learn more from negative errors and IR would learn more from positive (aversive SR + appetitive IR).

I did not find this argument to be convincing. In particular, the simulations of obsessive compulsive cycles (Fig. 2) take place in a punishment domain, yet despite this, the model used is appetitive SR + aversive IR. Additionally, the simulations demonstrate that aversive SR + appetitive IR in this domain (which they claim punishment domain might encourage) does not generate obsessive compulsive cycles. So, if I understood, the proposed model to explain the two-step task behavior is in conflict with the model used to explain obsessive compulsive cycles.

So, altogether, I'm somewhat uncertain about the extent to which the proposed model is supported as a model for OCD. The model of OCD decisions and variation from controls consists of two parts - 1) OCD participants use more IR component than healthy controls (both use an SR component), and 2) These components have different learning rates for positive and negative PEs, where SR is appetitive and IR is aversive. For the two-step task data, 1) is supported, but 2) is not. However, it's worth noting that 1) is not really a new prediction for this task. Because it is known that SR and IR can stand in for MB and MF, the new account is not really different from the standard account of this data, which is that there is a shift from MB to MF control in OCD.

In contrast, to generate the obsessive compulsive cycles, it is really 2) that is needed, but not 1). That is, the key feature needed to explain variation between healthy controls and OCD patients in developing OCD cycles is imbalanced learning rates, not differences in amount of IR in addition to SR. So, in this regard, if the point of the paper is to offer a new model of generation of obsessive compulsive cycles, I'm not sure that the two-step task data is really offering support to the key feature of that model that is needed to support these cycles.

Overall, I do think the basic observation that some phenomena (the simulations of obsessive compulsive cycles and the data of Sakai et al.) which previously were argued to support mechanism of imbalance eligibility traces, could also be explained by a combination SR + IR learner with imbalanced learning rates is interesting still. But I'm not really satisfied with how the falsification of, what I view as the key part of this model, in the two-step task data, is explained.

Thank you very much for your critical but important comments. Reading your comment and the other reviewer's comment, we have now come to think that the explanation of obsession-compulsion by the appetitive SR + aversive IR agent in the environmental model that we used (developed by Sakai et al.) would indeed has a significant difficulty. We have then realized that the aversive SR + appetitive

IR combination rather than the appetitive SR + aversive IR combination (as suggested by the two-stage task data) could instead specifically develop obsession-compulsion if the environment is modeled in a (rather slightly) different manner. We have described this in the new section of the Discussion ("An alternative environmental model"), which is copied in page 9~ of this document. We think that this alternative explanation could resolve the issues that you raised above.

Copy of the newly added section in the Discussion and Figure 10 of the revised manuscript

An alternative environmental model

The aversive IR + appetitive SR combination did not develop enhanced obsession-compulsion cycles in the environmental model that we used (proposed by [10]), as shown in the top-left corner of Figure 3E and Figure 4H. If OCD patients have both appetitive SR + aversive IR and aversive SR + appetitive IR combinations in the parallel circuits for reward RL and threat RL, respectively, as proposed in Figure 8, obsession-compulsion of the patients could still potentially be explained by the former combination which developed enhanced obsession-compulsion cycles. However, a difficulty with this possibility is whether the circuit for reward RL, rather than that for threat RL, could be activated in such an aversive situation where the patients develop obsession-compulsion. Or even more generally, overgeneralization of (or longer memory trace for) positive, rather than negative, feedback that causes enhanced obsession-compulsion cycles in this environmental model might not be intuitively convincing, and can actually be inconsistent with (even opposite to) the previous work examining generalization in OCD patients [50] as discussed above.

A different possibility, then, is that the aversive SR + appetitive IR combination, in the threat RL circuit, could in fact develop obsession-compulsion if the environment is modeled differently. Indeed, in an alternative environmental model shown in Figure 10A, the aversive SR + appetitive IR combination developed intermittent bursts of repetitive obsession-compulsions, whereas the other combinations (appetitive SR + aversive IR, neutral SR + neutral IR, SR only, and IR only) rarely did so (Figure 10B,C). In this alternative environmental model, "compulsion" causes a stay at the obsession state with punishment whereas "depart" causes a transition to the relief state without cost or punishment, and so rational agents should learn to avoid "compulsion". Likewise, "intrusive" causes a transition to the obsession state with large punishment whereas "normal" causes a stay at the relief state without punishment, and so "intrusive" should normatively be minimized. However, the aversive SR + appetitive IR agent could overgeneralize the large punishing feedback upon "intrusive" (entering the obsession state) to preceding "depart" and "normal", deteriorating their values and increasing, in turn, the probabilities that "compulsion" and "intrusive" are chosen. Key differences from the original environmental model (Figure 2, [10]) are (i) punishment is given upon entering the obsession state, not only upon staying at it, (ii) "compulsion" could be repeated without returning to the relief state every time, and (iii) intrusive thought, rather than "abnormal reaction" to it, is modeled. Given these features, this alternative environmental model not only demonstrates that the aversive SR + appetitive IR combination could develop obsession-compulsion but also potentially overcomes several difficulties in the original environmental model discussed above.

This being said, however, consideration of such an alternative environmental model may be ad hoc. The agents' behavioral patterns in this model have parameter dependence. Specifically, while moderately changing the size of punishment upon staying at the obsession state (Figure 10D, top two

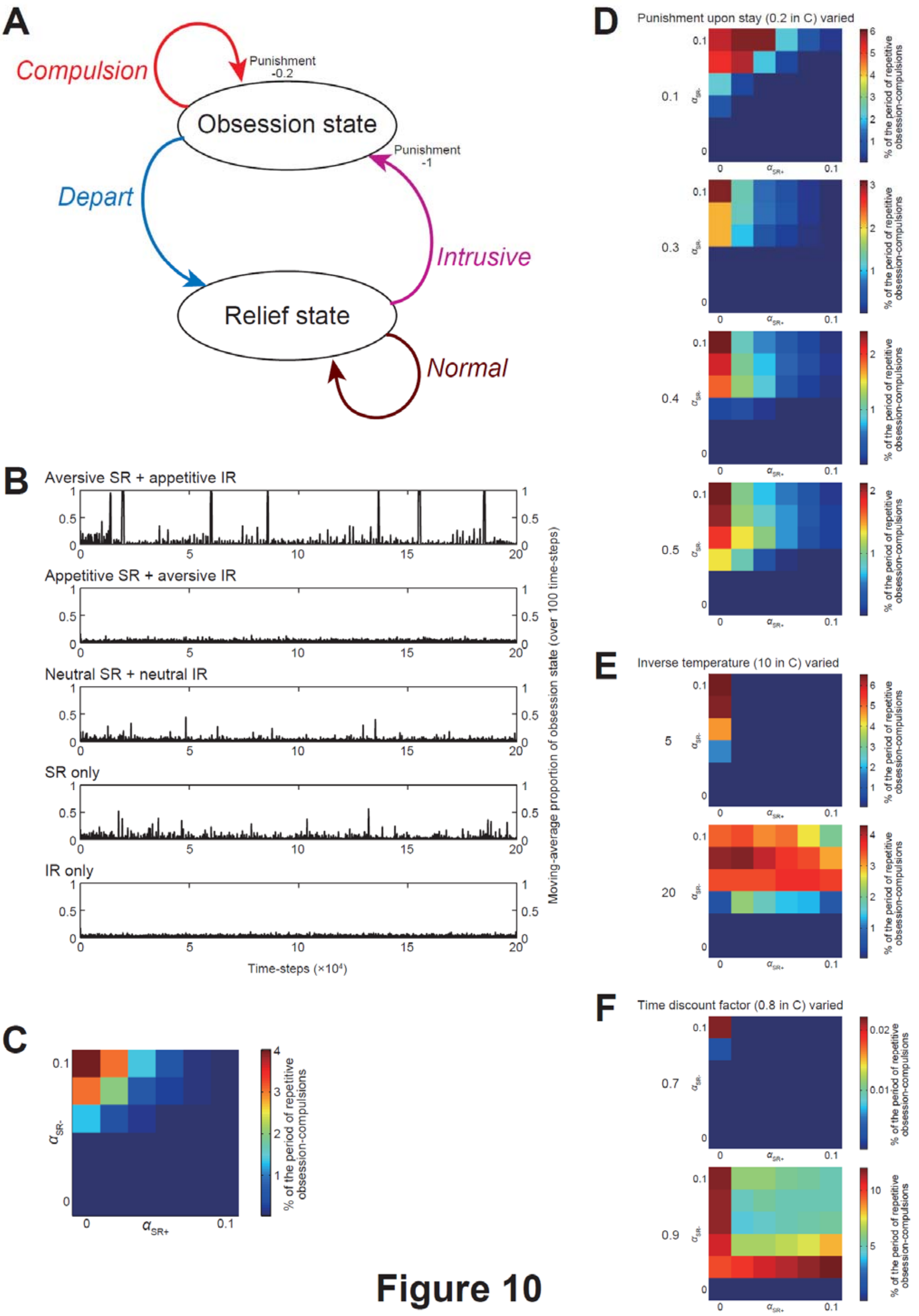
A
Compulsion
Punishment -0.2
Obsession state
Depart
Punishment -1
Intrusive
Relief state
Normal
B
Aversive SR + appetitive IR
Appetitive SR + aversive IR
Neutral SR + neutral IR
SR only
IR only
Time-steps (×10⁴)
Moving-average proportion of obsession state (over 100 time-steps)
C
% of the period of repetitive obsession-compulsions
D
Punishment upon stay (0.2 in C) varied
E
Inverse temperature (10 in C) varied
F
Time discount factor (0.8 in C) varied

Figure 10

Figure 10
Behavior of the dual-system agents in the alternative environmental model. **(A)** Diagram of action-state transitions. At the relief state, the agent can have "normal (thought)" or "intrusive (thought)". Having "intrusive" causes transition to the obsession state, with punishment. At the obsession state, the agent can take "compulsion", which causes a stay at the obsession state with punishment, or "depart", which causes transition to the relief state. **(B)** Examples of the moving-average proportion of the obsession state (averaged over 100 time-steps, plotted every 100 time-steps) in the cases of the different types of agents (from top to bottom: aversive SR + appetitive IR (α_{SR+}, α_{SR-}, α_{IR+}, α_{IR-}) = (0.01, 0.09, 0.09, 0.01), appetitive SR + aversive IR (0.09, 0.01, 0.01, 0.09), neutral SR + neutral IR (0.05, 0.05, 0.05, 0.05), SR-only (0.1, 0.1, 0, 0) and IR-only (0, 0, 0.1, 0.1)). **(C)** Percentage of the period of repetitive obsession-compulsions, in which the moving-average proportion of the obsession state was ≥ 0.5, during time-steps 0~50000 in various cases with different learning rates, averaged across 100 simulations for each case. The horizontal and vertical axes indicate α_{SR+} and α_{SR-}, respectively, while α_{SR+} + α_{IR+} and α_{SR-} + α_{IR-} were kept constant at 0.1 (in the same manner as in Figure 3E and Figure 4H). **(D-F)** Percentage of the period of repetitive obsession-compulsions in the cases where the size of punishment upon staying at the obsession state (originally 0.2 in (C)) was changed to 0.1, 0.3, 0.4, or 0.5 (D), the inverse temperature (originally 10 in (C)) was changed to 5 or 20 (E), or the time discount factor (originally 0.8 in (C)) was changed to 0.7 or 0.9 (F).

panels) did not drastically change the patterns, further increasing the punishment upon stay (Figure 10D, bottom), changing the inverse temperature (Figure 10E), or changing the time discount factor (Figure 10F) caused significant changes: disappearance of (or prominent decrease in) the intermittent bursts of repetitive obsession-compulsions in the aversive SR + appetitive IR combination and/or appearance of them in other combinations. These results might rather indicate a fundamental limitation of modeling the environment for psychiatric disorders like OCD by such simple diagrams with only a few states and actions (c.f., [58]). Nonetheless, we would like to argue that the parallel opponent SR+IR configurations, which can be in line with the differential cortical targeting/activations of the direct and indirect basal-ganglia pathways and the existence of parallel cortico-basal ganglia-dopamine circuits for reward RL and threat RL, could potentially provide a novel biologically grounded framework to integrate the different lines of behavioral findings on OCD: memory trace imbalance, impaired model-based control, and its valence-dependence.

Our trial on fitting of the choice data of Gillan et al. by our dual-system agent model

We fitted the choices of the participants with high OCI-R scores (≥40) in Experiment 1 (n = 23) and 2 (n = 58) of Gillan et al. (2016, *eLife*), whose choice patterns (stay probability depending on previous reward and transition) are shown in Figure F1A (same as Figure 9B), by our SR+IR dual-system model. As a result, for large parts of participants in both experiments, learning rate for positive RPEs was fitted to be not larger than that for negative RPEs in the SR system (i.e., $\alpha_{SR+} \leq \alpha_{SR-}$) whereas the opposite was the case in the IR system (i.e., $\alpha_{IR+} \geq \alpha_{IR-}$) (Figure F1B). We repeated the fitting analysis with the outcome encoded as no-punishment (0) or punishment (−1) instead of reward (1) or no-reward (0), and obtained similar results (Figure F1C). These results appeared consistent with the possibility that the participants with high OCI-R scores can be approximated by our aversive SR + appetitive IR agent.

However, we conducted the same fitting analyses using the choices of the participants with low OCI-R scores (≤1) in Experiment 2 (n = 79) of Gillan et al., whose choice pattern is shown in Figure F2A, and unexpectedly found that the fitting results looked largely similar to those for the high OCI-R participants (i.e., a large part of participants were fitted with $\alpha_{SR+} \leq \alpha_{SR-}$ and $\alpha_{IR+} \geq \alpha_{IR-}$). Given this, we cannot confidently argue that the fitted learning-rates actually well represent the participants' learning profiles. We checked if our fitting analysis could accurately reproduce parameter values from choices generated by simulations of the dual-system agent model itself. As a result, choices of appetitive SR + aversive IR agent were largely fitted with $\alpha_{SR+} > \alpha_{SR-}$ and $\alpha_{IR+} < \alpha_{IR-}$ (Figure F3A; largely around ($\alpha_{SR+} - \alpha_{SR-}$, $\alpha_{IR+} - \alpha_{IR-}$) = (0.24, −0.24) that was set in the simulation) whereas choices of aversive SR + appetitive IR agent were largely fitted with $\alpha_{SR+} < \alpha_{SR-}$ and $\alpha_{IR+} > \alpha_{IR-}$ (Figure F3B; largely around ($\alpha_{SR+} - \alpha_{SR-}$, $\alpha_{IR+} - \alpha_{IR-}$) = (−0.24, 0.24)), and the results for SR-only agent did not show either bias (Figure F3C; largely around ($\alpha_{SR+} - \alpha_{SR-}$, $\alpha_{IR+} - \alpha_{IR-}$) = (0, 0)). Thus, the bias in the fitted learning rates ($\alpha_{SR+} \leq \alpha_{SR-}$ and $\alpha_{IR+} \geq \alpha_{IR-}$) observed for the experimental data would imply a difficulty of fitting actual human choice data by this model. It might be due to the potential complexity of the model: even though the number of free parameters is not large (6), there are a number of fixed parameters, including the initial values of the elements of 6×6 SR matrix, potential differences in the learning rates (SR, IR, positive, negative) and inverse temperature between the first and second stages, and potential existence of eligibility traces etc. We noticed that previous work (Momennejad et al., 2017, *Nat Hum Behav*) that compared SR model and human choice behavior directly calculated the SR-based value by taking the inner product of fixed reward vector and SR features (row of SR matrix) rather than incorporating prediction error-based update, but such update is essential for our model.

Figure F1

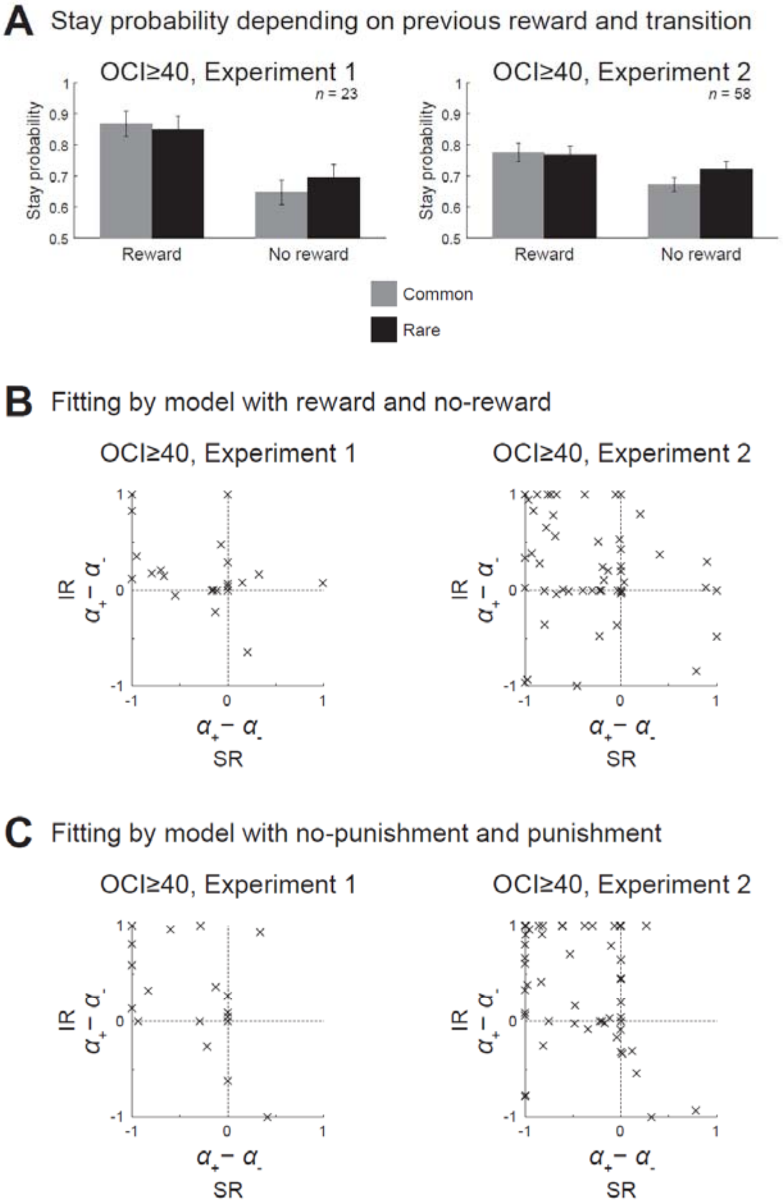

Figure F2

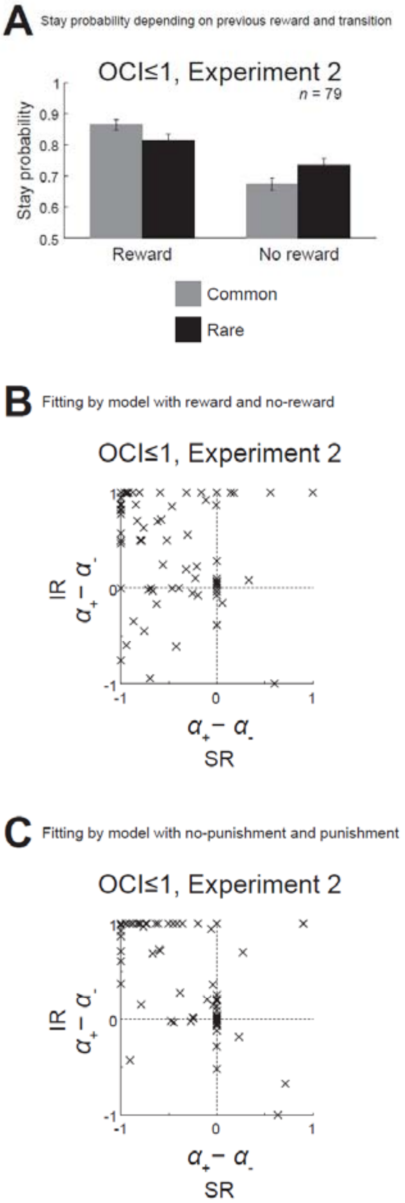

Figure F3

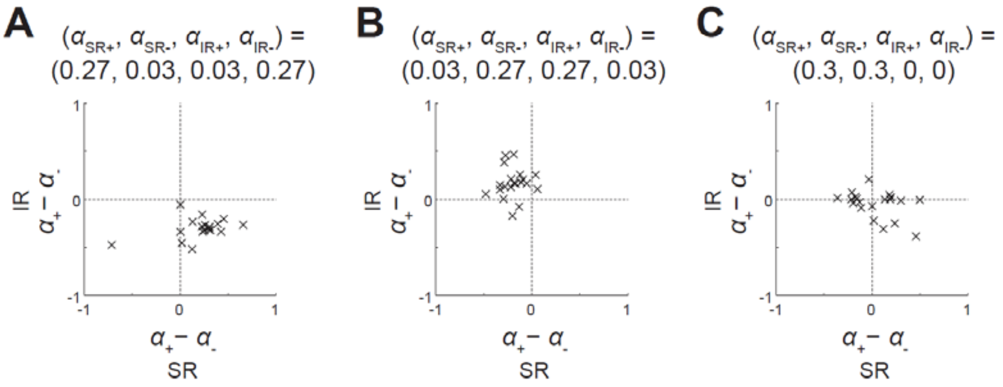

Supplement: Attachment — Submitted filename: ocd2rev2_responses_to_review_comments_v9.pdf [file pcbi.1011206.s003.pdf]
